# *PARK7*/DJ-1 deficiency impairs microglial activation in response to LPS-induced inflammation

**DOI:** 10.1186/s12974-024-03164-x

**Published:** 2024-07-16

**Authors:** Frida Lind-Holm Mogensen, Carole Sousa, Corrado Ameli, Katja Badanjak, Sandro L. Pereira, Arnaud Muller, Tony Kaoma, Djalil Coowar, Andrea Scafidi, Suresh K. Poovathingal, Maria Tziortziou, Paul M. A. Antony, Nathalie Nicot, Aurélien Ginolhac, Daniela M. Vogt Weisenhorn, Wolfgang Wurst, Aurélie Poli, Petr V. Nazarov, Alexander Skupin, Anne Grünewald, Alessandro Michelucci

**Affiliations:** 1 Neuro-Immunology Group, Department of Cancer Research, Luxembourg Institute of Health, 6A, rue Nicolas-Ernest Barblé, L-1210 Luxembourg, Luxembourg; 2 Faculty of Science, Technology and Medicine, University of Luxembourg, L-4365 Esch-sur-Alzette, Luxembourg; 3 Present Address: International Iberian Nanotechnology Laboratory, 4715-330 Braga, Portugal; 4 Integrative Cell Signalling Group, Luxembourg Centre for Systems Biomedicine, University of Luxembourg, L-4362 Esch-sur-Alzette, Luxembourg; 5 Molecular and Functional Neurobiology Group, Luxembourg Centre for Systems Biomedicine, University of Luxembourg, L-4362 Esch-sur-Alzette, Luxembourg; 6 Bioinformatics Platform, Department of Medical Informatics, Luxembourg Institute of Health, L-1445 Strassen, Luxembourg; 7 LuxGen Genome Center, Luxembourg Institute of Health and Laboratoire National de Santé, L-3555 Dudelange, Luxembourg; 8 Rodent Platform, Luxembourg Centre for Systems Biomedicine, University of Luxembourg, L-4362 Esch-sur-Alzette, Luxembourg; 9 Present Address: Single Cell Analytics and Microfluidics Core, Vlaams Instituut Voor Biotechnologie-KU Leuven, 3000 Louvain, Belgium; 10 Bioimaging Platform, Luxembourg Centre for Systems Biomedicine, University of Luxembourg, L-4362 Esch-sur-Alzette, Luxembourg; 11 Department of Life Sciences and Medicine, Faculty of Science, Technology and Medicine, University of Luxembourg, L-4365 Esch-sur-Alzette, Luxembourg; 12 Institute of Developmental Genetics, Helmholtz Zentrum München-German Research Center for Environmental Health, 85764 Neuherberg, Germany; 13 Technische Universität München-Weihenstephan, 85354 Freising, Germany; 14 German Center for Neurodegenerative Diseases (DZNE), 81377 Munich, Germany; 15 Munich Cluster for Systems Neurology (SyNergy), 81377 Munich, Germany; 16 Deutsche Zentrum für Psychische Gesundheit (DZPG), 80336 Munich, Germany; 17 Multiomics Data Science Group, Department of Cancer Research, Luxembourg Institute of Health, L-1445 Strassen, Luxembourg; 18 Department of Neuroscience, University of California San Diego, La Jolla, CA 92093 USA; 19 Integrative Biophysics, Department of Physics and Material Science, University of Luxembourg, L-1511 Luxembourg, Luxembourg; 20 Institute of Neurogenetics, University of Lübeck, 23538 Lübeck, Germany

**Keywords:** *PARK7*/DJ-1, Lipopolysaccharide, Microglia, Neuroinflammation, Parkinson’s disease, Microglia morphology

## Abstract

**Background:**

Specific microglia responses are thought to contribute to the development and progression of neurodegenerative diseases, including Parkinson’s disease (PD). However, the phenotypic acquisition of microglial cells and their role during the underlying neuroinflammatory processes remain largely elusive. Here, according to the multiple-hit hypothesis, which stipulates that PD etiology is determined by a combination of genetics and various environmental risk factors, we investigate microglial transcriptional programs and morphological adaptations under *PARK7*/DJ-1 deficiency, a genetic cause of PD, during lipopolysaccharide (LPS)-induced inflammation.

**Methods:**

Using a combination of single-cell RNA-sequencing, bulk RNA-sequencing, multicolor flow cytometry and immunofluorescence analyses, we comprehensively compared microglial cell phenotypic characteristics in *PARK7*/DJ-1 knock-out (KO) with wildtype littermate mice following 6- or 24-h intraperitoneal injection with LPS. For translational perspectives, we conducted corresponding analyses in human *PARK7*/DJ-1 mutant induced pluripotent stem cell (iPSC)-derived microglia and murine bone marrow-derived macrophages (BMDMs).

**Results:**

By excluding the contribution of other immune brain resident and peripheral cells, we show that microglia acutely isolated from *PARK7*/DJ-1 KO mice display a distinct phenotype, specially related to type II interferon and DNA damage response signaling, when compared with wildtype microglia, in response to LPS. We also detected discrete signatures in human *PARK7*/DJ-1 mutant iPSC-derived microglia and BMDMs from *PARK7*/DJ-1 KO mice. These specific transcriptional signatures were reflected at the morphological level, with microglia in LPS-treated *PARK7*/DJ-1 KO mice showing a less amoeboid cell shape compared to wildtype mice, both at 6 and 24 h after acute inflammation, as also observed in BMDMs.

**Conclusions:**

Taken together, our results show that, under inflammatory conditions, *PARK7*/DJ-1 deficiency skews microglia towards a distinct phenotype characterized by downregulation of genes involved in type II interferon signaling and a less prominent amoeboid morphology compared to wildtype microglia. These findings suggest that the underlying oxidative stress associated with the lack of *PARK7*/DJ-1 affects microglia neuroinflammatory responses, which may play a causative role in PD onset and progression.

**Supplementary Information:**

The online version contains supplementary material available at 10.1186/s12974-024-03164-x.

## Background

Microglia serve as the central nervous system’s primary immune effector cells. Under threatening conditions, microglia rapidly react by undergoing transcriptional, morphological and functional changes, known as “microglia activation”. This process encompasses various stages and substates, which vary depending on the context and duration of the compromising conditions, seeking to resolve the threat efficiently [52].

Parkinson’s disease (PD) is the second most common neurodegenerative disease, which involves the accumulation and aggregation of α-synuclein in the form of Lewy bodies and the progressive loss of dopaminergic neurons. The main risk factor for PD is aging and, as the world´s population becomes older, the number of people diagnosed with a neurodegenerative disease continues to increase [17]. Additionally, factors beyond aging, such as pesticide exposure and infections, may contribute significantly to this trend, as the incidence of PD currently outpaces the rate of aging and is disproportionally on the rise, a tendency that is particularly noticeable in newly industrialized countries [17]. The notion that PD is caused by a combination of genetics and different environmental risk factors refers to the multiple-hit hypothesis [53]. Among the detected genetic defects, the loss of function of *PARK7*, encoding for the protein DJ-1, causes autosomal recessive early-onset PD in humans. DJ-1 is a multifunctional protein with roles in cellular transformation, transcriptional regulation, and anti-oxidative stress functions [14]. Although *PARK7* mutations leading to DJ-1 loss of function are rare and GWAS studies failed to show a link between *PARK7* promoter polymorphisms and PD risk so far [31], they are causative of Lewy body pathology and α-synucleinopathy [69]. Further, low DJ-1 protein levels and over-oxidized forms have been detected in the cerebrospinal fluid, blood, urine and brain of idiopathic PD patients, suggesting that DJ-1 deficiencies may play a role in a larger spectrum of PD patients [13, 35, 42, 48, 57, 58]. On the other hand, among the environmental risk factors, a number of bacterial and viral infections, including *Helicobacter pylori* [8, 50] and hepatitis C virus [51], have been linked to increased idiopathic PD risk, thus indicating a potential contributing role of infections in the development or exacerbation of the disease, at least in certain individuals [20, 64, 80].

Activated microglial phenotypes and neuroinflammatory processes are detected in the early stages of PD and in the *substantia nigra* of post-mortem tissues [24, 34, 39, 63]. However, the role of microglia in the onset and progression of PD remains vague. In line with the multi-hit hypothesis, we here postulate that *PARK7*/DJ-1 deficiency influences microglial responses under inflammatory conditions. To address this hypothesis, we took advantage of the *PARK7*/DJ-1 KO mouse model, which shows subtle impairments in cognitive and motor functions and heightened vulnerability to neurotoxins [10, 26, 37]. How microglia react in those mice under inflammatory conditions has been addressed in specific models, while the majority of the studies have been conducted in vitro using *PARK7*/DJ-1 deficient immortalized microglial cell lines or primary cells, which show an exacerbated response to inflammatory stimuli [42]. By comparing the transcriptional profile of sorted microglial cells isolated from *PARK7*/DJ-1 KO with wildtype mice peripherally injected with lipopolysaccharide (LPS), a stimulus we have previously shown to induce a prominent microglia activation [67], we identified distinct signatures of this activated state. Specifically, microglia in *PARK7*/DJ-1 KO LPS-treated mice were characterized by the downregulation of genes involved in immune responses, especially interferon-related genes, and upregulation of genes related to DNA damage response, when compared to the corresponding cells isolated from wildtype mice. We further confirmed a dampened transcriptional response towards LPS in human *PARK7*/DJ-1 mutant compared to isogenic control induced pluripotent stem cell (iPSC)-derived microglia and murine bone marrow-derived macrophages. Furthermore, in line with these distinct profiles detected at the transcriptional level, microglial cells in *PARK7*/DJ-1 KO mice exhibited a less prominent amoeboid shape when compared to wildtype mice in response to LPS-induced systemic inflammation.

Taken together, our results show that, under inflammatory conditions, *PARK7*/DJ-1 deficiency skews microglia towards a distinct phenotype, which may have an impact during neuroinflammatory processes under threatening conditions, such as during α-synuclein accumulation at the onset and during the progression of PD.

## Materials and methods

### Mice

*PARK7*/DJ-1 KO (B6;129P2-*Park7*^*Gt(XE726) Byg*^/Mmucd) mice were kindly provided by the laboratory of Dr D.M. Vogt Weisenhorn in Munich (Germany), where they have been originally developed and characterized [55]. The *PARK7*/DJ-1 KO line used in our laboratory had already been backcrossed with C57BL/6NCrl mice for at least ten generations. For all the described experiments, we used *PARK7*/DJ-1 KO and wildtype littermate mice that were 3- and 4-month age-matched siblings, with the exception of the aged cohort where we used 12- and 13-month old mice, generated from heterozygous breeding pairs. Mice were bred and housed in a specific pathogen-free animal research facility at a relative humidity of 40–70%, at 22 °C and in 12 h light/dark cycles. Mice received food (SAFE A40) and water ad libitum.

### PARK7/DJ-1 KO mouse genotyping

*PARK7*/DJ-1 KO, heterozygote and wildtype littermates were genotyped using a genotyping kit (KAPA Biosystems, KK7352), following the manufacturer’s instructions. Briefly, genomic DNA was extracted from mouse ear marking samples or tail pieces. The PCR cycling program (BioRad 3000) was as follows: 1 cycle of initial denaturation at 95 °C for 3 min followed by 35 cycles of denaturation at 95 °C for 15 s, annealing at 58 °C for 15 s and extension at 72 °C for 15 s, ended by a final extension at 72 °C for 7 min. Primers used for genotyping were as follows. Wildtype *Park7* forward primer: 5’-AGGCAGTGGAGAAGTCCATC-3’; wildtype *Park7* reverse primer: 5’-AACATACAGACCCGGGATGA-3’; KO *Park7* reverse primer: 5’-CGGTACCAGACTCTCCCATC-3’. PCR products loaded on a 2% agarose gel showed bands at 475 bp or 231 bp, corresponding to wildtype or KO alleles, respectively.

### Acute microglia isolation by fluorescence-activated cell sorting (FACS)

Mice were treated with a single intraperitoneal injection of LPS (4 μg/g body weight) or with PBS (saline) as vehicle control for 24 h. Processing of the brain, microglial isolation and multicolor flow cytometry preparation was done as previously described [67]. Briefly, myelin was removed from cell suspension with the Myelin removal kit (130-096-733, Miltenyi Biotec), according to the manufacturer’s protocol. For multicolor staining, cells were incubated for 15 min with Fc receptor binding inhibitor to reduce the binding of non-specific Fc-gamma receptors, and then stained with fluorochrome-conjugated antibodies or their corresponding isotype controls for 45 min at 4 °C in the dark (Table S1). After washing, cells were pelleted at 300 g for 10 min at 4 °C and resuspended in 200 µL of the appropriated buffer. Hoechst (0.1 µg/ml; Sigma) or Sytox Red (1:1000; Thermo Fisher Scientific) were added shortly before flow cytometry measurements for dead cell discrimination. CD11b^+^CD45^int^ living single cells were sorted with FACS Aria™ SORP cytometer (BD Biosciences) fitted with a 640 nm (30 mW) red laser, a 355 nm (60 mW) UV laser, a 405 nm (50 mW) violet laser, a 488 nm (100 mW) blue laser and a 561 nm (50 mW) yellow/green laser. Data were analyzed with FACSDiva (Becton Dickinson) and FlowJo (v7.6.5; Tree Star) software.

### Single-cell RNA-sequencing using Drop-seq, bioinformatics and data analyses

FACS-sorted CD11b^+^CD45^int^ cells were collected in pre-cooled HBSS, 0.5% BSA and transferred directly for subsequent Drop-seq analysis as previously described [67].

#### Cell calling, normalization and scaling

DropletUtils [44] was employed to distinguish cells from empty droplets. Subsequently, raw gene counts of the identified “*cells*” were converted into Seurat object [30]. Cells expressing less than 100 genes and genes detected in less than 3 cells were discarded. The resulting object was subjected to log-normalization and scaling using the standard functions of the Seurat package (*NormalizeData and ScaleData;* Seurat v4.3.0.1)*.*

#### Dimension reduction and clustering

Principal component analysis was performed via singular value decomposition (function *propack.svd*, svd package, v0.5.4). Correlations between each principal component (PC) and technical/experimental factors, such as the total number of counts per cell and mouse genotype, were estimated using linear models. Given the strong correlation between the first PC and the total number of counts per cell, we selected PCs 2 to 20 for uniform manifold approximation and projection (UMAP) and clustering analysis. UMAP was generated using the *RunUMAP* function of the Seurat package, while cells were clustered using *FindNeighbors* function followed by *FindClusters*.

#### Differential expression analysis

Differential expression analysis was conducted using the MAST algorithm [21] as implemented in the *FindAllMarkers* or *FindMarkers* functions of the Seurat package. *FindAllMarkers* was employed to identify marker genes specific to each cell cluster while *FindMarkers* was used for other comparative analyses.

### CD11b magnetic enrichment for ex vivo analyses

Mice were treated with a single intraperitoneal injection of lipopolysaccharide (LPS) (Sigma, *E.Coli* O55:B5, L6529-1MG) (4 μg/g body weight) or with PBS (saline) as vehicle control for 6 h for ex vivo analyses. Mice were terminally anaesthetized with a combination of Ketamine (100 mg/mL; Nimatek Vet) / Dorbene (medetomidine / hydrochloride; 1 mg/mL; Dorbene vet) and perfused transcardially with PBS. Brains were rapidly dissected and stored in PBS on ice. The olfactory bulb and cerebellum were removed and the brain dissected into small pieces in HBSS (Lonza) in a petri dish. Brain pieces were collected in HBSS, centrifuged and resuspended in an enzymatic mix, following the manufacturer’s instructions for the neural dissociation kit (130-092-628, Miltenyi Biotec). After dissociation, cell suspensions were incubated for 20 min with CD11b beads (130-049-60, Miltenyi Biotec) and passed through LS columns (130-042-401, Miltenyi Biotec) to enrich microglia by magnetic separation. Lastly, CD11b^+^ cells were collected and either stained for flow cytometry analyses or treated with RLT lysis buffer for subsequent RNA extraction using the Qiagen RNA isolation kit following the manufacturer’s instructions (RNeasy® Mini Kit, 74,104, Qiagen).

### Human induced pluripotent stem cell-derived microglia

Induced pluripotent stem cells (iPSCs) carrying the c.192G > C mutation in *PARK7* causing the amino acid change p.E64D in DJ-1 and leading to aberrant splicing [7, 38] were cultured in mTeSR™ Plus complete medium (Stem Cell Technologies) in 6-well plates. Embryoid bodies (EBs) were generated from iPSCs by supplementing mTeSR™ medium with specific growth factors (Table S2). On day 4, EBs were transferred to T75 flasks to form “factories” [76]. The iPSCs were differentiated into microglia following an established protocol [28, 76]. See Table S2 for medium composition and growth factors/supplements for each differentiation step. Cells were kept at 37 °C in a humidified incubator at 5% CO_2_. Microglia-like cells were cultured in 6-well plates at a density of 1 × 10^6^ cells/well and treated with 100 ng/mL LPS (Sigma, *E.Coli* O55:B5, L6529-1MG) for 6 h. RNA was extracted using the Qiagen RNA isolation kit following the manufacturer’s instructions (RNeasy® Mini Kit, 74,104 Qiagen).

### Bone marrow-derived macrophage differentiation and treatments

After euthanasia, tibia and femur from *PARK7*/DJ-1 KO mice and wildtype littermates were dissected out and kept in ice-cold DMEM Glutamax (61960526, Gibco) for 1.5-12 h before the extraction of bone-marrow cells (BMCs). Both extremities of tibia and femur were cut to allow a 26 g needle to flush BMCs according to standardized protocols [74]. The cell pellet was treated with NH4Cl (0.17 M) for 5 min to remove red blood cells. BMCs were cultured on low-attachment conditions in DMEM Glutamax (61965026, Gibco), supplemented with 10% FBS (10270-106, Gibco), 1% penicillin-streptavidin (DE17-602E, Lonza), 1 mM sodium pyruvate (11360070, Gibco) and 20 ng/mL M-CSF (416-ML-010/CF, Biotechne). The complete medium was supplemented on day 4. On day 8, fully differentiated bone marrow-derived macrophages (BMDMs) were treated with LPS (100 ng/mL) for 6 h. To investigate their morphological features, BMDMs were stained with Acti-stain™ 488 Fluorescent Phalloidin (PHDG1, Cytoskeleton, Inc.) and incubated for 1.5 h at room temperature at day 8 of differentiation with or without prior LPS treatment. Actin cytoskeleton was imaged with a Nikon Eclipse microscope at a 10 × magnification and analyzed using CellProfiler™ [68].

### Primary microglia and treatments

CD11b^+^ cells were collected as previously described. Primary adult microglia were plated overnight in 48-well plates coated with poly-L-lysine (0.1 mg/mL solution; Sigma-Aldrich) at a density of 3 × 10^5^ cells per well and grown in DMEM-F12 supplemented with 10% FBS (Life Technologies) and pen-strep (100 U/mL / 100 μg/ml; Life Technologies). Cells were stimulated with interferon-gamma (IFNγ; 315-05, Peprotech) at a final concentration of 50 ng/mL for 6 h.

### RNA sequencing and bioinformatics analyses

Cells were pelleted down and lysed in RLT buffer for subsequent RNA extraction using the Qiagen RNA isolation kit following the manufacturer’s instructions (RNeasy® Mini Kit, 74104, Qiagen). A DNase treatment followed by a clean-up has been added using the RNA Clean & Concentrator™-5 kit (Zymo Research) following the manufacturer’s instructions. Quantification of total RNA was performed using a N60 Nanophotometer® (Implen GmbH) and a Qubit 2.0 Fluorometer® (Thermo Fisher Scientific) with the Qubit RNA HS Assay Kit. The quality was assessed using a Fragment Analyzer System (Agilent Technologies) using a RNA Pico Chip (Agilent Technologies) (only samples with RQN ≥ 7 were further analyzed). The Illumina® stranded Total RNA Prep, Ligation with Ribo-Zero Plus kit Library preparation has been used to process libraries for RNA-sequencing according to Illumina’s Reference Guide # 1000000124514 v00 June 2020, starting with 25 ng of total RNA. All libraries have been quantified using a Qubit HS dsDNA kit (Thermo Fisher Scientific) and the library quality check has been performed using a High Sensitivity NGS Fragment Analysis Kit on the Fragment Analyzer System (Agilent Technologies). Indexed libraries were normalized and pooled to be sequenced on an Illumina Novaseq 6000 sequencer according to manufacturer’s instructions with targeted conditions of 2 × 75 basepair and 25 M reads/sample. For the murine dataset, raw fastq files were quality checked using a combination of *FastQC*, *fastq_screen* and *RSeQC* tools wrapped in *MultiQC v1.11* [19] followed by a preliminary analysis composed of read trimming, mapping and counting using *Cutadapt* [45], STAR 2.7.9a [16] and the Mouse genome (GRCm39 – Mus_musculus.GRCm39.108.gtf). For the human dataset, raw fastq files were quality checked using a combination of *FastQC* and *fastq_screen* tools wrapped in *MultiQC v1.11* [19]. Reads were trimmed using *AdapterRemoval v2.3.2* [60], and mapped against the human genome (Ensembl GRCh38.p14) with the splice-aware STAR 2.7.9a [16]. Resulting alignments were processed by *featureCounts* (package RsubRead v2.8.1) [40] to obtain gene counts further used by *DESeq2* for differential gene expression. All of these steps were integrated into a local *snakemake* [49] environment. The template for the human dataset is available in release 0.3.2: https://gitlab.lcsb.uni.lu/aurelien.ginolhac/snakemake-rna-seq/-/releases/v0.3.2. The software set used by the template on the ULHPC [77] was bundled in a Docker image available at https://hub.docker.com/r/ginolhac/snake-rna-seq tag v0.5. Differential gene expression analysis was conducted using R statistical software v4.2.3 [72] and DESeq2 v1.38.3 for the murine dataset and v1.42.0 for the human dataset [43] using the resulting gene count tables. Heatmaps were created using *ComplexHeatmap* v2.18.0 [27].

### Gene ontology analysis

Differentially expressed genes were submitted to Database for Annotation, Visualization and Integrated Discovery (DAVID) [62] for further exploration of Gene Ontology (GO) terms for biological processes (BP) using overrepresentation analysis. Additionally, we run gene set enrichment analysis (GSEA) by R package *clusterProfiler (v.4.10.1)* using log fold changes from the group comparisons for gene ordering.

### Reverse transcription and qPCR

Reverse transcription was performed using SuperScript™ III (18080093, Invitrogen) according to the manufacturer’s instructions. At first, oligo (dT) primers (50 μM) and 5 mM dNTP mix were added to the RNA and incubated at 65 °C for 5 min. Subsequently, tubes were incubated at 4 °C for 1 min and then 5 × first-strand buffer, 0.1 M DTT (707265ML, Thermo Fisher Scientific), RNase OUT (10777019, Thermo Fisher Scientific) and 200 units/μl SuperScript™ III RT (18080051, Thermo Fisher Scientific) were added and incubated for 45 min at 50 °C. The reaction was lastly inactivated by heating for 15 min at 70 °C. The qPCRs were carried out in 384-well plates (Applied Biosystems QuantStudio5 Real-Time PCR System). All samples were run in three technical triplicates. Diluted cDNA was mixed with 10 μL of Fast SYBR Green Master Mix (Applied Biosystems) and 0.5 μL of 10 μM gene-specific forward and reverse primers. All primers were designed using the Primer BLAST tool with the following parameters: Tm 60 °C ± 3 °C, possibly spanning exon-exon junctions separated by at least one intron on the corresponding genomic DNA region and a product size between 75 and 300 bp (Table S3). The qPCRs were performed using the following standardized program: 20 s at 95 °C followed by 35 cycles of PCR stage of 1 s at 95 °C (denaturation) and 20 s at 60 °C (annealing) and a melt curve stage of 15 s at 95 °C, 1 min at 60 °C and 10 s at 95 °C. The average threshold cycle (Ct) values were used to quantify the mRNA product by the ΔΔCt method normalized to the housekeeping gene *Gapdh*, which showed better stability than other housekeeping genes (e.g. *Rpl27*) across experimental groups (data not shown).

### Protein extraction and Western blotting

Protein samples were prepared by lysing whole brain tissues or Magnetic-Activated Cell Sorted (MACS)-CD11b^+^ cells from wildtype and *PARK7*/DJ-1 KO mice, human fibroblasts and iPSCs in cell lysis buffer (Pierce RIPA, 89900, Thermo Fisher Scientific), supplemented with Halt protease and phosphatase inhibitor (78444, Thermo Fisher Scientific). Protein extracts were then normalized, denatured at 95–100 °C for 6–8 min, resolved on a 4–12% BisTris gel (NP0323, Lifetech), transferred on polyvinylidene difluoride (PVDF) membranes (LifeTech) and blocked with 2% milk in Tris-buffered saline (TBS) solution containing 0.1% Triton X-100. Anti-DJ-1 (Cell Signaling) was used for protein detection, while anti-α-actin (Merck Millipore) was used for signal normalization. Details of these antibodies can be found in Table S1. After washes, the membranes were incubated either with HRP-conjugated secondary antibodies or Rabbit IgG Cross-Adsorbed Secondary Antibody, DyLight™ 800 (SA5-10036, Thermo Fisher Scientific), according to the manufacturer’s instructions. Signal development was performed either via addition of Super Signal West Femto Maximum Sensitivity Substrate (Thermo Fisher Scientific) followed by image acquisition with ImageQuant LAS4010 imaging station (HRP-conjugated secondary antibodies) or via Odyssey Fc Imaging System (LI-COR Biosciences) (Rabbit IgG Cross-Adsorbed Secondary Antibody, DyLight™ 800).

### Cytoplasmic and mitochondrial reactive oxygen species measurements

#### Bone marrow-derived macrophages

BMDMs were differentiated as previously described and plated in 96-well plates high performance #1.5 cover glass (0.170 ± 0.005 mm) (Cellvis) at a density of 2 × 10^5^ cells per well. Cells were treated with LPS (L4391, E. Coli O111:B4, Sigma-Aldrich) at a final concentration of 100 ng/mL at day 7 for overnight treatment or for 6 h on the day of imaging (day 8 of differentiation). Cells were plated to have 3 technical replicates per condition.

#### Primary microglia

Primary microglia were obtained as previously described and cultured for less than 24 h before imaging in 96-well plates coated with poly-L-lysine (1 μg/mL solution; Sigma-Aldrich) at a density of 1.5 × 10^5^ cells per well. Two hours after seeding, microglia were treated with LPS (100 ng/mL) overnight. Cells were plated to have 2 technical replicates per condition.

To assess the mitochondrial membrane potential, cells were incubated with 150 µL/well of full medium with TMRE (T669, Life Technologies) at a final concentration of 10 nM for 30 min. Cells were co-stained with Hoechst 33342 (62249, Life Technologies) at 1 µM, MitoTracker Green-FM (M7514, Life Technologies) at 100 nM to derive the mitochondrial mask, and CellROX Deep Red (C10422, Life Technologies) at 5 µM to assess oxidative stress levels. Medium was subsequently removed and cells were washed twice following incubation in medium containing 10 nM of TMRE. Images were acquired using the Yokogawa CellVoyager CV8000 High-Content Screening System, a spinning-disk confocal microscope, using a 60 × water immersion objective. The following bandpass filters were used: BP 445–45, BP 525–50, BP 600–37, and BP 676–29. Z-stacks were acquired with a slicing interval of 0.4 µm and 12 or 13 fields per well were obtained for BMDMs and microglia respectively. Automated segmentation of diverse structures (i.e. nuclei, mitochondria and CellRox Deep Red area) and the subsequent feature extraction, which included mitochondrial morphometrics, was performed using Matlab (version 2021a, MathWorks) by adapting a previously used method [2]. The analysis was run on the High Performance Computing (HPC) facilities of the University of Luxembourg in collaboration with the LCSB-Bioimaging platform. The code can be shared upon request.

### Tissue preparation for immunohistochemistry analyses

#### Cryosectioning preparation (6-h LPS time point)

After perfusion with cold PBS, one brain hemisphere per mouse was drop-fixed in 4% paraformaldehyde (PFA, Lonza) for 24 h, washed with PBS and immersed in 30% sucrose (Sigma, SO389) in PBS until complete permeation. When the brain was at the bottom of the tube, it was cut via a rodent brain matrix (ASP Instruments, RBM-2000C) into 4 sagittal macro-sections per hemisphere, which were kept at − 80 °C until further processing. Subsequently, the macro-sections were embedded in OCT (Tissue-Tek) and cut on a cryostat into 50 μm sections. Tissue sections were mounted on glass slides (Epredia Superfrost™ Plus Adhesion, Epredia™ J1800AMNZ) and washed in PBS.

#### Floating section preparation (24-h LPS time point)

After perfusion with cold PBS, the brain was drop-fixed in 4% PFA for 48 h. Subsequently, brains were stored at 4 °C in 0.02% sodium azide/PBS and serialized parasagittal free floating 30 µm-thick sections were generated with a vibratome (Leica; VT-1000S) and collected in cryoprotective medium, PBS containing 1–1 ethylene glycol (Sigma-Aldrich) and 1% w/v polyvinylpyrrolidone (Sigma-Aldrich). Sections were stored at − 20 °C until further processing.

### Immunofluorescence staining

Tissue sections were incubated in 3% H_2_O_2_ + 1.5% Triton X-100 for 30 min and subsequently washed with PBS and blocked in 5% BSA for 30 min. Tissue sections were incubated with primary antibodies at room temperature overnight and washed with PBS before incubating them with secondary antibody in 2% BSA 0.3% Triton X-100 for 2 h at RT (Table S1). After washing three times with PBS for 10 min, tissue sections were mounted using mounting medium containing 4ʹ,6-diamidino-2-phenylindole (DAPI) (Invitrogen, 00–4959-52) and covered with glass coverslips. Images were acquired using a LSM 880 confocal microscope (Zeiss). iPSCs were plated on coverslips and fixed in 4% PFA for 10 min, washed with PBS three times for 5 min, subsequently permeabilised and blocked in 3% BSA and 0.4% Triton-X-100 for 1 h, washed in PBS and incubated overnight with primary antibodies (Table S1) in 1% BSA and 0.25% Triton-X-100. Finally, iPSCs were incubated for 2 h with secondary antibodies and Hoechst and mounted on glass slides.

### Image acquisition and application of microglia and immune-cells morphological analysis and clustering (MIC-MAC) 2

Stained slides were imaged on a LSM 880 confocal microscope (Zeiss) with the 20 × objective (laser and filters) (the experimenter was blinded for genotype and treatment during acquisition). Nine 3D confocal tile scans were captured with a resolution of 0.221 μm in the x and y dimensions and a resolution of 1 μm in the z dimension. The average thickness per 3D image was 30 µm, and between 28 and 31 μm, depending on the antibody penetration. The resulting 3D stacks of IBA1^+^ microglial cells were inspected with Imaris and filtered to ensure coherent and high standards of image quality. The 20 × 3D images were then processed with Imaris where slice intensities were corrected using the *Normalizer Layer* function, followed by Gaussian Filter (width = 0.3). Segmentation of IBA1^+^ cells was performed in Imaris by using the *Surface Creation* Tool (*Local Contracts* with *Smooth* enabled and surface detail of 0.5 μm) with a threshold manually adjusted to account for small variations in the contrast of the images (threshold parameter between 0.5 and 1). The *Split Touching Object* option was disabled, and the resulting 3D structures were filtered by size (between 18,000 and 180,000 voxels) to filter out incomplete structures or microglia clusters that would inevitably bias the morphological analysis. For the 24-h LPS time point, images were captured using a 40 × objective with a voxel size of: 0.415 × 0.415 × 0.524 μm^3^ with the 405 nm and 633 nm lasers with a pinhole of 42.6 = 1.47 Airy unit (1 μm section). Digital gain DAPI: 1, gain master 860. Offset − 2. A647, Digital gain: 1, Gain master 846 and offset − 7. The morphological feature extraction of each segmented cell was ultimately performed by using *Microglia and Immune-cells Morphological Analysis and Clustering (MIC-MAC) 2*, which allows for automatic segmentation of microglia from 3D fluorescent microscopy images [22]. The mice included for these analyses were as follows: wildtype control group: 2 males and 2 females; wildtype LPS group: 1 male and 2 females; *PARK7*/DJ-1 KO control group: 1 male and 2 females; *PARK7*/DJ-1 KO LPS group: 2 males and 2 females.

### Statistical analyses

Data was analyzed in Excel and Prism v10.0.3. For qPCR data, fold change from control condition or 2^−ΔCT^ between groups was calculated and compared via ANOVA with multiple comparisons. Comparison of two groups of normally distributed data was performed using unpaired t-tests. If data did not pass tests for normality using Kolmogorov–Smirnov (alpha = 0.05), a non-parametric Kruskal–Wallis test was performed followed by Dunn’s test for multiple comparisons. All experiments included at least 3 biological replicates. All tests were performed on a significance level of 5% and all graphs depict means with standard error of the mean (SEM).

## Results

### Distinct activation profiles detected by single-cell transcriptional analysis of acutely isolated CD11b^+^CD45^int^ microglial cells between wildtype and *PARK7*/DJ-1 KO mice 24 h after LPS-induced systemic inflammation

**Fig. 1:**
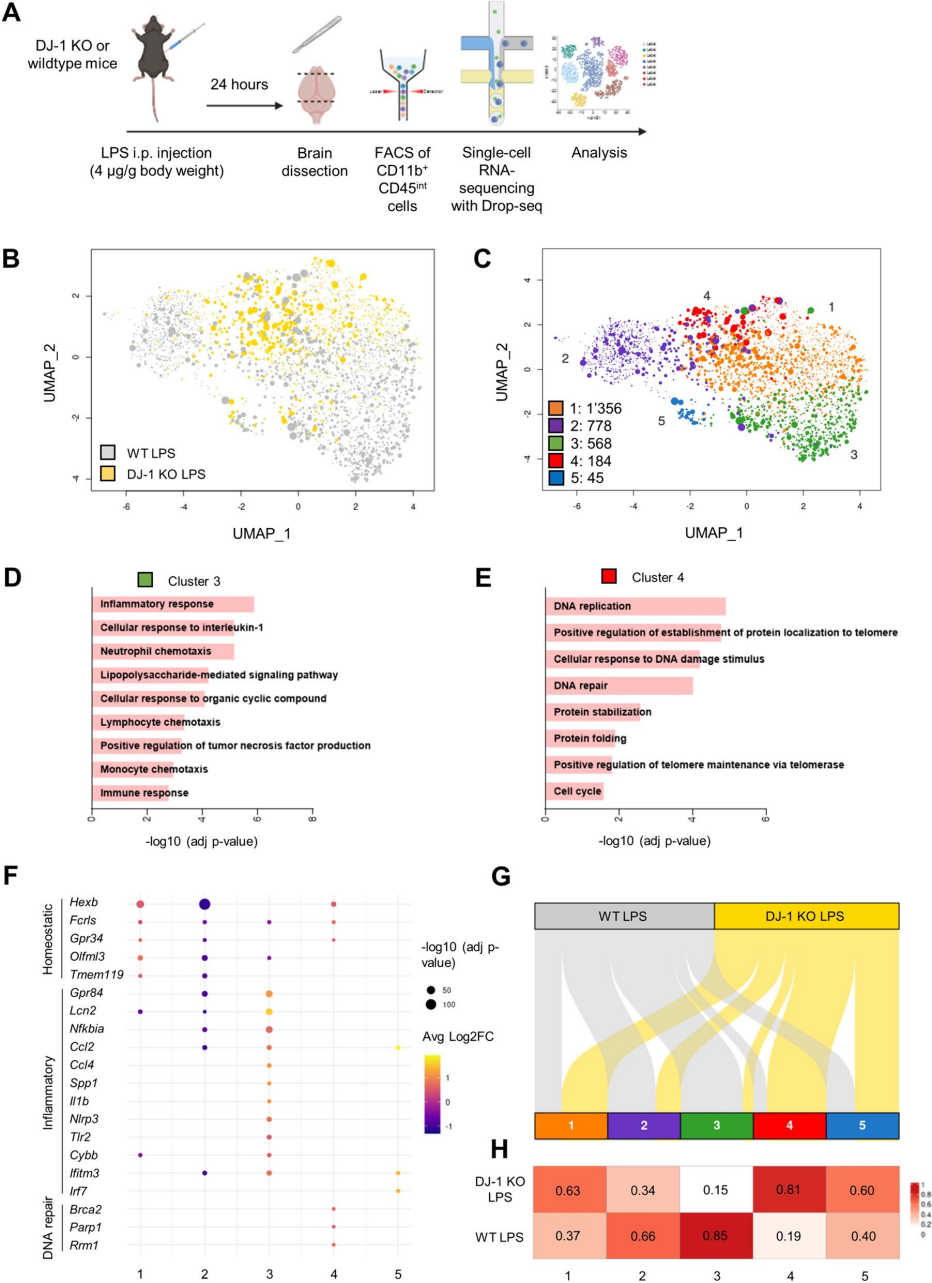
Subset of classical activated microglia is mainly composed of cells originated from wildtype mice 24 h after systemic LPS-induced inflammation. **A** Schematic representation of scRNA-sequencing analyses of CD11b^+^CD45^int^ microglial cells isolated either from *PARK7*/DJ-1 KO or wildtype mice 24 h following LPS treatment. **B** UMAP showing 2931 CD11b^+^CD45^int^ microglial cells from *PARK7*/DJ-1 KO (yellow) and wildtype (grey) mice 24 h after LPS treatment (n = 2 mice per group). The different size of the dots in the UMAP represents the “read count per cell”, with large or small dots representing cells with high or low read count, respectively. **C** UMAP showing clustering analysis of 2931 single microglial cells. Numbers indicate amount of cells per cluster. **D**, **E** Gene ontology terms corresponding to upregulated genes (adjusted p-value < 0.05, log2FC ≥ 0.5) comparing (**D**) Cluster 3 or (**E**) Cluster 4 to the other clusters. **F** Dot plot showing selected differentially expressed genes (adjusted p-value < 0.05, |log2FC|≥ 0.5) characterizing each cluster. Color code represents the average log2 Fold Change (FC), while the size of the dot is proportional to the statistical significance indicated as − log10 (adjusted p-value). **G** Alluvial plot showing CD11b^+^CD45^int^ microglial cells flowing into the identified 5 normalized clusters according to their origin, either from wildtype (grey) or *PARK7*/DJ-1 KO (yellow) mice. **H** Heatmap showing percentages of cells deriving either from *PARK7*/DJ-1 KO or wildtype mice

First, we confirmed the lack of DJ-1 in the brain of the corresponding KO mice. As expected, while DJ-1 was expressed in the brain tissue of wildtype mice, it was neither detected in brain tissue nor in CD11b^+^CD45^int^ microglia isolated from *PARK7*/DJ-1 KO mice (Fig. S1A). In our previous study, we demonstrated that intraperitoneal injection of LPS at a dosage of 4 μg/g body weight in C57BL/6N mice led to a significant activation of microglial cells after 24 h [67]. To investigate the transcriptional profile of microglia under the same inflammatory conditions in mice lacking *PARK7*/DJ-1, we FACS-isolated CD11b^+^CD45^int^ cells, previously identified as predominantly representing microglia [67], from both wildtype and *PARK7*/DJ-1 KO mice after 24 h of LPS injection. Immuno-profiling of the brain under these conditions showed increases in the levels of monocytes (CD11b^+^CD45^+^Ly6C^+^ cells) and decreases in the amount of lymphocytes (CD11b^−^CD45^+^ cells) compared to baseline conditions, indicative of established neuroinflammation at similar levels both in wildtype and *PARK7*/DJ-1 KO mice (Fig. S1B,C). We conducted single-cell RNA-sequencing of CD11b^+^CD45^int^ cells using the Drop-seq technology (Fig. 1A) and performed subsequent clustering analysis of 2931 microglial cells, with 953 cells isolated from two *PARK7*/DJ-1 KO mice and 1978 cells from two wildtype mice. This approach enabled us to identify five distinct clusters in the corresponding UMAPs (Fig. 1B,C). To verify that the clustering analysis was not driven by the sex of the mouse of origin, we discriminated female and male cells based on the expression levels of X-inactive specific transcript (*Xist*), a female cell-specific nuclear long noncoding RNA crucial for X-chromosome inactivation [5, 15]. The partition of the cells in “*Xist* high” and “*Xist* low” enabled to discriminate cells, respectively from females (536 cells from *PARK7*/DJ-1 KO mouse and 781 cells from wildtype mouse) and males (417 cells from *PARK7*/DJ-1 KO mouse and 1197 cells from wildtype mouse), with cells from both sexes grouping across the five clusters, suggesting that our analysis is independent from the mouse sex (Fig. S1D). After identifying differentially expressed genes (DEGs) that characterize each cluster compared to the others, we conducted gene ontology (GO) term enrichment analysis by submitting the up-regulated DEGs from each cluster (adjusted p-value < 0.05, log2FC ≥ 0.5) to DAVID (Table S4). The resulting GO term for Cluster 1 included “synapse disassembly”, while Cluster 2 was characterized by terms related to transcriptional regulation, such as “RNA splicing”, “chromatin organization” and “mRNA processing” (Fig. S1E). Cluster 3 was defined by GO terms related to classical inflammatory responses, including “cellular response to interleukin-1” and “lipopolysaccharide-mediated signalling pathway”, with upregulation of microglial pro-inflammatory markers (e.g. *Gpr84*, *Lcn2* and *Nfkbia*) (Fig. 1D), while Cluster 4 was characterized by terms linked to DNA damage response, such as “DNA replication” and “DNA repair”, with overexpression of homeostatic microglial genes (e.g. *Hexb*, *Fcrls* and *Gpr34*) and DNA repair markers (e.g. *Brca2*, *Parp1* and *Rrm1*) (Fig. 1E). Lastly, Cluster 5 was associated with GO terms related to host defense responses, primarily against viruses, including “defense response to virus” and “cellular response to interferon-beta” (Fig. S1E) (Table S4). We showed a selection of DEGs (considering adjusted p-value < 0.05, |log2FC|> 0.5) characterizing each cluster in a dot plot (Fig. 1F). When examining the composition of each cluster based on the amount of cells per mouse genotype using an alluvial plot (Fig. 1G) and analyzing the corresponding ratios using a heatmap (Fig. 1H), we observed that clusters 1, 2 and 5 were relatively equally represented in both genotypes. In contrast, Cluster 3, associated with classical inflammatory responses, was predominantly constituted by microglial cells from wildtype mice (85%), whereas Cluster 4, linked to DNA damage response and cell cycle, primarily consisted of cells originating from *PARK7*/DJ-1 KO mice (81%) (Fig. 1G,H). We excluded the presence of proliferating microglial cells in *PARK7*/DJ-1 KO mice by analyzing the expression of KI67 in IBA1^+^ microglial cells in cortical brain slices as well as gene expression levels of *Mki67* in CD11b^+^CD45^int^ microglial cells isolated from the brain of *PARK7*/DJ-1 KO and wildtype mice after 24 h of LPS injection. KI67 was not detectable at the protein level (Fig. S1F) and barely expressed at the gene level (Fig. S1G).

Taken together, these results show that microglia in wildtype mice display a classical inflammatory profile following 24 h of LPS injection, characterized by substates associated to transcriptional regulation (Cluster 2) and pro-inflammatory responses (Cluster 3), while microglia in *PARK7*/DJ-1 KO mice show an enrichment in DNA damage response signatures (Cluster 4), suggesting a potential involvement in repair and tolerance mechanisms.

### Diverse transcriptional activation profiles of microglia detectable after 6 h of peripheral LPS-induced inflammation in *PARK7*/DJ-1 KO compared to wildtype mice

**Fig. 2:**
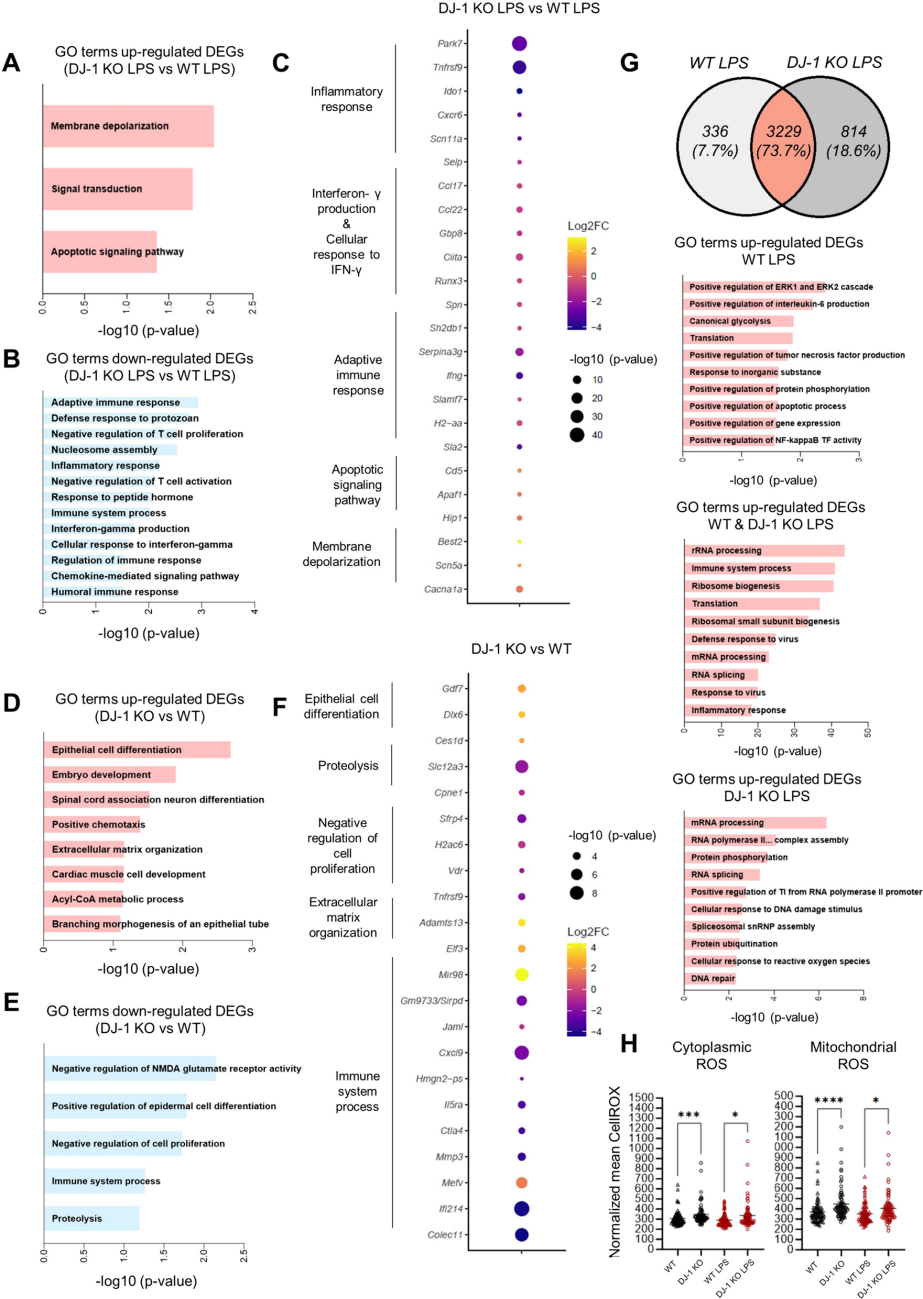
Distinct reaction of microglia in *PARK7*/DJ-1 KO mice compared to wildtype mice 6 h after systemic LPS-induced inflammation and at baseline. **A**, **B** GO terms corresponding to **A** upregulated or **B** downregulated genes comparing microglia in *PARK7*/DJ-1 KO and wildtype male mice 6 h after LPS treatment. **C** Dot plot showing a selection of differentially expressed genes (p-value < 0.05, |log2FC|≥ 0.5) comparing CD11b^+^ microglia isolated from *PARK7*/DJ-1 KO and wildtype mice 6 h after LPS-induced inflammation. Color bar shows log2 Fold Change (FC), while the size of the dot is proportional to the statistical significance indicated as − log10 (p-value). **D**–**E** GO terms corresponding to **D** upregulated or **E** downregulated genes comparing microglia in *PARK7*/DJ-1 KO and wildtype mice at baseline. **F** Dot plot showing a selection of variable genes (p-value < 0.05, |log2FC|≥ 0.5) comparing CD11b^+^ microglia isolated from *PARK7*/DJ-1 KO and wildtype mice at baseline. Color bar shows log2 Fold Change (FC), while the size of the dot is proportional to the statistical significance indicated as –log10 (p-value). **G** Venn diagram and GO terms corresponding to upregulated genes matching differentially expressed genes (adjusted p-value < 0.05, log2FC ≥ 0.5) resulting from the comparison of microglia in wildtype and *PARK7*/DJ-1 KO mice 6 h after LPS treatment. **H** Cytoplasmic and mitochondrial ROS levels in microglia isolated from wildtype and *PARK7*/DJ-1 KO male mice cultured overnight left untreated or treated with LPS (100 ng/mL) and analyzed by live imaging. Graphs show normalized CellROX mean of analyzed images (dots). Kruskal–Wallis test with multiple comparisons. ****p < 0.0001; ***p < 0.001; *p < 0.05 (n = mean of 26 images per condition from 3 mice per group)

Next, we sought to investigate whether the distinct microglial profiles identified after 24 h of LPS-induced inflammation were also apparent at earlier time points. With this analysis, we aimed to discern if the impaired microglial reactivity under *PARK7*/DJ-1 deficiency manifested during the early phases of the inflammatory response or if they indicated premature resolution substates. For this, we intraperitoneally injected *PARK7*/DJ-1 KO (n = 5) and wildtype (n = 5) mice with LPS (4 μg/g body weight) and MACS-isolated CD11b^+^ cells from dissected brains after 6 h for subsequent transcriptional analyses (Fig. S2A). We verified the validity of our sorting protocol by analyzing the expression levels of oligodendrocyte (*Mog* and *Mobp*), astrocyte (*Gfap* and *Ntsr2*) and neuronal (*Tubb3* and *NeuN*) markers in MACS-isolated CD11b^+^ cells compared to the negative fraction. Those markers were undetectable or only expressed at very low levels in cells collected within the positive fraction, which on the contrary expressed high levels of the microglial markers (*Gpr34* and *Olfml3*) (Fig. S2B). We tested the purity of CD11b^+^ cells by staining plated sorted cells with IBA1 antibody and detected 90–97% (mean 94.5% ± 1.9%) of IBA1^+^ cells among CD11b^+^ cells, thus indicating an enrichment of microglial cells (Fig. S2C). By conducting RNA-sequencing analyses of MACS-isolated CD11b^+^ cells from *PARK7*/DJ-1 KO and age-matched wildtype mice following 6 h of LPS injection, we detected 474 DEGs (p-value < 0.05, |log2FC|≥ 0.5) comparing KO versus wildtype. Among them, 227 genes were upregulated, while 247 genes were downregulated (Table S5). GO terms resulting from the 227 upregulated DEGs comparing CD11b^+^ cells isolated from *PARK7*/DJ-1 KO and wildtype mice in response to LPS were “membrane depolarization”, “signal transduction” and “apoptotic signaling pathway” (Fig. 2A). On the other hand, GO terms resulting from the corresponding 247 downregulated DEGs were linked to the immune response, mainly related to the regulation of the adaptive immune system, such as “adaptive immune response” and “negative regulation of T cell proliferation”, and interferon-gamma-related pathways, exemplified by “interferon-gamma production” and “cellular response to interferon-gamma” terms (Fig. 2B). Among the related down-regulated genes, *Tnfrsf9, Ciita, Serpina3g, Ifng* and *H2.Aa* were in the top 50 protein-coding DEGs (Fig. 2C).

The characterization of these distinct profiles under LPS treatment prompted us to investigate whether differences between microglial cells in *PARK7*/DJ-1 KO and wildtype mice were already detectable at baseline. Through RNA-sequencing analysis of MACS-isolated CD11b^+^ cells from *PARK7*/DJ-1 KO (n = 5) and age-matched wildtype (n = 4) mice, we detected 308 DEGs (p-value < 0.05, |log2FC|≥ 0.5) comparing KO versus wildtype. Among them, 103 genes were upregulated, while 205 genes were downregulated (Table S5). GO terms resulting from the 103 upregulated DEGs comparing CD11b^+^ cells isolated from *PARK7*/DJ-1 KO and wildtype mice at baseline were mainly related to developmental processes and morphological adaptation, such as “epithelial cell differentiation”, “extracellular matrix organization” and “branching morphogenesis of an epithelial tube” (Fig. 2D), while terms resulting from the corresponding 205 downregulated DEGs included differentiation and immune response (Fig. 2E). Among the related down-regulated genes, *Cxcl9* and *Ifi214*, linked to the interferon-gamma signaling, were in the top 50 protein-coding DEGs (Fig. 2F). We confirmed the decrease of the expression levels of these genes and the increase of *Mefv* by qPCR including additional CD11b^+^ brain cell samples derived from independent male and female mice (Fig. S2D). In line with reported results showing an exaggerated response of *PARK7*/DJ-1 deficient microglia towards pro-inflammatory stimuli in vitro, microglia isolated from *PARK7*/DJ-1 KO treated with IFNγ (50 ng/mL) for 6 h showed increased expression levels, albeit not statistically significant, of *Cxcl9* and *Ifi214* compared with cells isolated from wildtype mice (Fig. S2E), suggesting that potential compensatory mechanisms taking place in the brain milieu are not recapitulated when microglia are isolated and cultured in vitro. Further, in contrast with the results we obtained in adult mice, the expression levels of pro-inflammatory markers, including *Il6* and *Cxcl9*, were up-regulated in MACS-isolated CD11b^+^ microglial cells from *PARK7*/DJ-1 KO aged mice (12–13 month old) compared to age-matched wildtype mice treated with LPS (4 μg/g body weight) for 6 h (Fig. S2F), indicating that compensatory mechanisms underlying the distinct activation profile in adult mice are possibly exhausted with aging.

By conducting a direct comparison of the LPS-induced genetic responses between microglia isolated from wildtype and *PARK7*/DJ-1 KO adult mice, we identified DEGs comparing LPS and baseline conditions in microglia (adjusted p-value < 0.05, |log2FC|≥ 0.5), both from wildtype and KO mice (Table S6), and compared the detected genes. Most of the identified genes (3229 DEGs, corresponding to 73.7% of total DEGs) were up-regulated in both LPS-treated conditions and, as expected, were mainly associated with GO terms linked to inflammatory responses. On the other hand, GO terms corresponding to 336 unique up-regulated DEGs in microglia from wildtype mice were associated with classical microglial activation, such as “positive regulation of tumor necrosis factor production” and “positive regulation of NF-kappaB TF activity”, while GO terms related to 814 unique up-regulated DEGs in microglia from *PARK7*/DJ-1 KO mice, such as “cellular response to DNA damage stimulus”, were linked to DNA repair processes (Fig. 2G). Thus, these results corroborate the findings obtained by scRNA-seq analyses after 24 h of LPS treatment. Further, as “cellular response to reactive oxygen species” was among the terms associated with up-regulated DEGs in microglia from *PARK7*/DJ-1 KO mice, we measured the levels of microglial reactive oxygen species (ROS). For this, we isolated CD11b^+^ microglial cells from *PARK7*/DJ-1 KO and wildtype mice, cultured overnight with or without LPS (100 ng/mL) and subsequently assessed ROS levels and mitochondrial membrane potential with live imaging. As expected, ROS tracer intensity in the cytoplasm and mitochondria was higher in microglia from *PARK7*/DJ-1 KO compared with wildtype mice, both at baseline and following LPS treatment (Fig. 2H). Although there were no differences in mitochondrial membrane potential between microglia from *PARK7*/DJ-1 KO and wildtype mice at baseline, it was decreased in LPS-treated samples, with a significantly stronger loss of mitochondrial membrane potential in microglia from *PARK7*/DJ-1 KO mice (Fig. S2G), indicating that increased ROS amounts in these cells induce higher levels of mitochondrial clearance to possibly sustain their viability.

At the protein level, in line with the detected transcriptional dampened immune activation of microglia in *PARK7*/DJ-1 KO compared to wildtype adult mice treated with LPS, we detected decreased expression levels of CD11b (Fig. S2H).

Taken together, these results show that microglia in *PARK7*/DJ-1 KO adult mice downregulate genes associated with inflammatory responses, specially linked to interferon-gamma signaling, both at the early stages of inflammatory conditions and at baseline, compared to wildtype mice. On the other hand, microglia in *PARK7*/DJ-1 KO mice treated for 6 h with LPS upregulate genes related to “membrane depolarization”, “signal transduction” and “apoptotic signalling pathway”, which, in line with the described functions of DJ-1 as an important protector against ROS and the DNA damage response signatures identified after 24 h of LPS treatment by scRNA-sequencing (Cluster 4), are related to repair and tolerance mechanisms, such as attenuation of mitochondrial reactive oxygen species generation [56].

### Discrete activated transcriptional profile of microglia derived from iPSCs harboring the c.192G > C mutation in *PARK7* compared to isogenic controls 6 h after LPS treatment

Next, to investigate whether these findings hold true in the human context, we applied a translational approach to verify if the microglial transcriptional phenotype identified in *PARK7*/DJ-1 KO mice was similar in human *PARK7*/DJ-1 mutant iPSC-derived microglia. Briefly, iPSCs carrying the c.192G > C mutation in *PARK7* causing the amino acid change p.E64D in DJ-1 were previously described to lead to undetectable levels of DJ-1 protein in these cells due to aberrant splicing [7]. Both isogenic control and mutant iPSCs expressed similar levels of the typical pluripotency markers, including stage-specific embryonic antigen-4 (SSEA4), podocalyxin (TRA-1-60), octamer-binding transcription factor 4 (OCT4) and sex determining region-Y box (SOX2), thus suggesting that *PARK7*/DJ-1 deficiency does not affect their expression (Fig. S3A,B). As expected, while we measured high levels of *PARK7*/DJ-1 expression in isogenic control fibroblasts and iPSCs, both at the protein and mRNA level, its expression was not detectable in the corresponding mutant cells (Fig. S3C,D). We derived microglial cells from iPSCs following an established protocol [28, 76]. Analysis of *PARK7* in the resultant iPSC-derived microglia (iMG) confirmed the lack of its expression in the mutant cells (Fig. S3C). Gene expression analyses of three human microglia homeostatic genes, *GPR34*, *TREM2* and *P2RY12*, in both isogenic control and mutant cultures, showed increased expression levels in iMG when compared to undifferentiated iPSCs, thus indicating effective differentiation of the two lines into microglia-like cells (Fig. S3E).

**Fig. 3:**
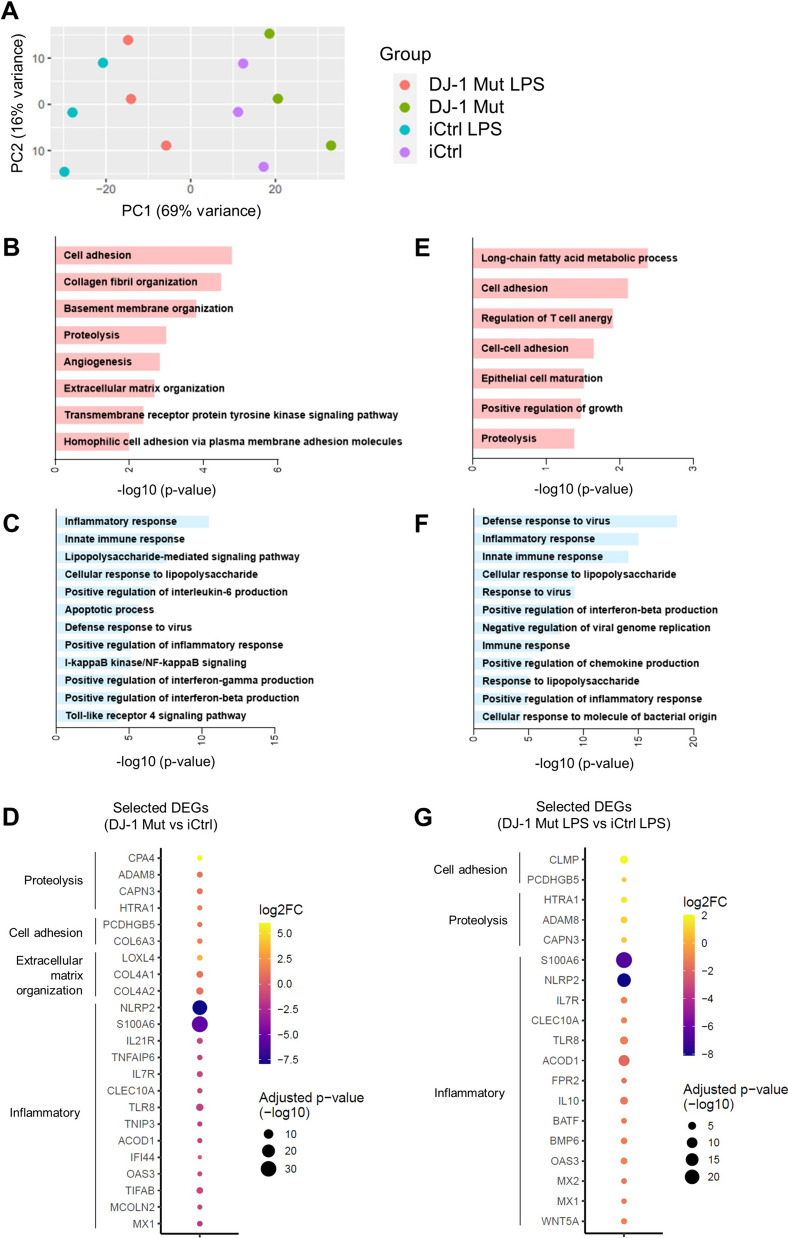
Discrete iPSC-derived microglia signatures between *PARK7*/DJ-1 mutant and isogenic control cells under LPS treatment and at baseline. **A** Design aware Principal component analysis (PCA) plot showing clustering of 3 replicates of differentiated iPSC-derived microglia (iMG) per condition: *PARK7*/DJ-1 mutant and isogenic control iMG untreated or treated with LPS for 6 h. **B-C** GO terms corresponding to **B** upregulated or **C** downregulated genes comparing *PARK7*/DJ-1 mutant and isogenic control iMG at baseline. **D** Dot plot showing selected differentially expressed genes (adjusted p-value < 0.05, |log2FC|≥ 0.5) comparing *PARK7*/DJ-1 mutant and isogenic control iMG at baseline. Color bar shows log2 Fold Change (FC) and the size of the dot is proportional to the statistical significance indicated as –log10 (adjusted p-value). **E–F** GO terms corresponding to **E** upregulated or **F** downregulated genes comparing *PARK7*/DJ-1 mutant and isogenic control iMG after 6 h LPS treatment. **G** Dot plot showing selected differentially expressed genes (adjusted p-value < 0.05, |log2FC|≥ 0.5) comparing *PARK7*/DJ-1 mutant and isogenic control iMG after treatment with LPS for 6 h. Color bar shows log2 Fold Change (FC) and the size of the dot is proportional to the statistical significance indicated as –log10 (adjusted p-value)

As conducted in the mouse model, we carried out RNA sequencing to compare the transcriptional signatures of *PARK7*/DJ-1 mutant and isogenic control iMG, both with or without a LPS (100 ng/mL) treatment for 6 h, which resulted in relatively distinct clusters according to the four conditions (Fig. 3A). At baseline, among 197 DEGs (adjusted p-value < 0.05, |log2FC|≥ 0.5) (Table S7), we detected 103 upregulated genes in *PARK7*/DJ-1 mutant iMG. GO terms associated to those upregulated genes included “cell adhesion”, “proteolysis” and “extracellular matrix organization” (Fig. 3B). By contrast, GO analysis of the 94 downregulated genes comprised terms linked to immune responses, such as “cellular response to lipopolysaccharide” and “positive regulation of interferon-gamma production” (Fig. 3C), thus indicating an effect on microglial immune surveillance and activation already at baseline. We showed a selection of corresponding up- (e.g., *CPA4, PCDHGB5, LOXL4*) and down- (e.g., *NLRP2, S100A6, ACOD1, IFI44*) regulated genes (adjusted p-value < 0.05, |log2FC|≥ 0.5) characterizing *PARK7*/DJ-1 mutant iMG in a dot plot (Fig. 3D).

Under LPS conditions, we detected 289 DEGs (adjusted p-value < 0.05, |log2FC|≥ 0.5), when comparing *PARK7*/DJ-1 mutant and isogenic control iMG (Table S7). Among them, 83 genes were upregulated, while 206 were downregulated in *PARK7*/DJ-1 mutant iMG. GO analyses of up-regulated genes showed terms associated with long-chain fatty acid metabolism and cell adhesion (Fig. 3E), while corresponding analyses of down-regulated genes resulted in terms related to immune responses, including “defense response to virus”, “cellular response to lipopolysaccharide” and “positive regulation of interferon-beta production” (Fig. 3F). We displayed a selection of resulting up- (e.g., *CLMP, PCDHGB5, HTRA1, ADAM8*) and down-regulated (e.g., *S100A6, NLRP2, ACOD1*) genes (adjusted p-value < 0.05, |log2FC|≥ 0.5) characterizing *PARK7*/DJ-1 mutant iMG treated with LPS using a dot plot (Fig. 3G). Interestingly, some of these genes were commonly up- (e.g., *CLMP, HTRA1, ADAM8*) or down-regulated (e.g., *ACOD1, S100A6, NLRP2*) both at baseline and LPS conditions comparing *PARK7*/DJ-1 mutant and isogenic control iMG (Fig. S3F).

Overall, these results show that *PARK7*/DJ-1 mutant iMG, in line with the analogous murine cells, display an attenuated response to LPS compared to corresponding isogenic control cells. We further verified this analogy by analyzing GO terms in common between *PARK7*/DJ-1 mutant iMG and corresponding murine cells under LPS conditions, which resulted in 23 shared GO terms (Fig. S4A) associated to a dampened immune response in both species (Fig. S4B).

### Enhanced compactness at baseline and less pronounced amoeboid morphology of microglia in *PARK7*/DJ-1 KO compared to wildtype mice after 6 and 24 h of LPS treatment

Since the identified distinct microglial gene expression patterns may represent morphological phenotypes evident in brain tissues, we investigated microglial morphological changes in *PARK7*/DJ-1 KO and wildtype mice at baseline and under LPS conditions. To this end, we stained brain tissues with an antibody against the microglial marker ionized calcium-binding adapter molecule 1 (IBA1) and applied MIC-MAC 2 to the resulting 3D confocal images in order to segment and classify microglial cells based on their morphology [22, 59]. While, homeostatic surveying microglia were highly ramified with multiple processes (nodes), activated microglia show a more amoeboid and compact shape. Using MIC-MAC 2, we were able to extract morphological features based on the shape, structure and size of microglia, enabling to score the cells based on the levels of their roundness or elongation.

**Fig. 4:**
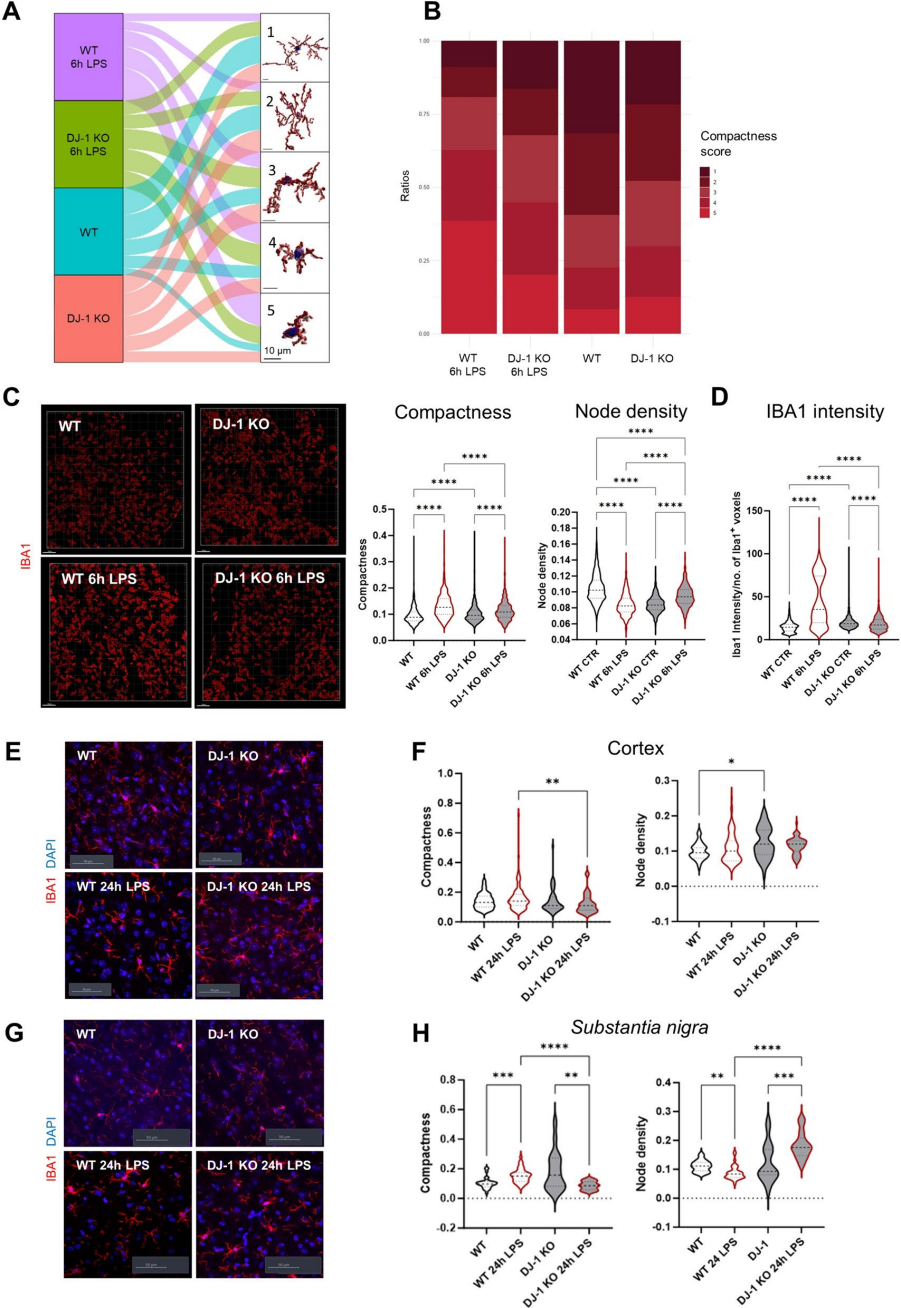
Microglia in *PARK7*/DJ-1 KO mice show a more compact morphology compared to wildtype mice at baseline and a less amoeboid morphology 6 and 24 h after LPS treatment. **A** Alluvial plot showing MIC-MAC analysis of IBA1^+^ cortical microglial cells flowing into 5 morphological clusters (pictures show 3D reconstructions of a representative microglial cell per cluster, scale bar: 10 μm) based on compactness score (spanning from cluster 1, corresponding to homeostatic ramified microglia, to cluster 5, representing amoeboid cells) according to their mouse origin, either from wildtype (baseline in turquoise, 6 h LPS in purple) or *PARK7*/DJ-1 KO (baseline in red, 6 h LPS in green) (n = 3–4 mice). **B** Bar graph showing the ratios of the identified clusters across the four different conditions (wildtype or *PARK7*/DJ-1 KO 6 h LPS, wildtype or *PARK7*/DJ-1 KO baseline) (mean = 244 ± 19 cells per cortex region from 3–4 mice per group). Color bar shows compactness score. **C** Left: representative pictures showing 3D reconstruction of cortical IBA1^+^ cells in *PARK7*/DJ-1 KO or wildtype mice with or without 6 h LPS treatment. The shade of red indicates the cluster to which the cell belongs. Scale bar: 150 μm. Right: violin plots showing mean compactness (left) or node density (right) (dotted line) of cortical microglial cells in wildtype or *PARK7*/DJ-1 KO mice with or without 6 h LPS treatment. Kruskal–Wallis test with Dunn’s test for multiple comparisons. ****p < 0.0001 (mean = 244 ± 19 cells per cortex region from 3–4 mice per group). **D** Violin plot showing mean of IBA1 intensity (dotted line) divided by the number of IBA1^+^ voxels of cortical microglial cells in wildtype or *PARK7*/DJ-1 KO mice with or without 6 h LPS treatment. Kruskal–Wallis test with Dunn’s test for multiple comparisons. ****p < 0.0001 (mean = 244 ± 19 cells per cortex region from 3–4 mice per group). **E** Representative pictures showing cortical IBA1^+^ cells in *PARK7*/DJ-1 KO or wildtype mice with or without 24 h LPS treatment. Scale bar: 50 μm. **F** Violin plots showing mean compactness (left) or node density (right) (dotted line) of cortical microglial cells in wildtype or *PARK7*/DJ-1 KO mice with or without 24 h LPS treatment. Kruskal–Wallis test with Dunn’s test for multiple comparisons. **p < 0.01, *p < 0.05 (mean = 22 ± 2 cells per cortex region from 3 mice per group). **G** Representative pictures showing *substantia nigra* IBA1^+^ cells in *PARK7*/DJ-1 KO or wildtype mice with or without 24 h LPS treatment. Scale bar: 50 μm. **H** Violin plots showing mean compactness (left) or node density (right) (dotted line) of *substantia nigra* microglial cells in wildtype or *PARK7*/DJ-1 KO mice with or without 24 h LPS treatment. Kruskal–Wallis test with Dunn’s test for multiple comparisons. ****p < 0.0001, ***p < 0.001, **p < 0.01 (mean = 9 ± 1 cells per substantia nigra region from 3 mice per group)

Within the analyzed cortical regions, we recognized five distinct morphological clusters that were differentially represented in *PARK7*/DJ-1 KO and wildtype mice at baseline and after 6 h of LPS-treatment (Fig. 4A). Corresponding quantifications showed decreased ratios of ramified microglia (Cluster 1) and enhanced proportions of amoeboid microglia (Cluster 5) in *PARK7*/DJ-1 KO compared with wildtype mice at baseline (Fig. 4B). Under LPS conditions, the ratio of amoeboid microglia (Cluster 5) was increased compared to baseline conditions, although this rise was less prominent in *PARK7*/DJ-1 KO compared with wildtype mice (Fig. 4B). We further confirmed these results by coupling the analysis of relevant features, including compactness and node density, across the various conditions. Indeed, cortical microglia were more amoeboid and less ramified (fewer nodes) at baseline, while they were less amoeboid and more ramified after 6 h LPS-treatment in *PARK7*/DJ-1 KO compared with wildtype mice (Fig. 4C). In line with these observations, the intensity of IBA1 expression per number of IBA1^+^ voxels was higher in wildtype compared with *PARK7*/DJ-1 KO brains 6 h after LPS treatment (Fig. 4D). By similarly investigating morphological changes of microglia 24 h following LPS treatment, we detected higher compactness and lower node density of cortical microglia in wildtype compared to *PARK7*/DJ-1 KO mice (Fig. 4E,F). This trend was even more evident in the *substantia nigra*, a brain region that is primarily affected in PD (Fig. 4G,H), indicating a more prominent effect of *PARK7*/DJ-1 KO deficiency in microglial morphological adaptation in PD-related brain regions.

Taken together, our results show that microglia in *PARK7*/DJ-1 deficient mice display different morphologies at baseline and exhibit a distinct morphological adaptation in response to LPS-induced inflammation compared to wildtype microglia after 6 and 24 h of systemic LPS treatment.

### Type II interferon-related gene impairment, prominent compactness at baseline and less evident amoeboid morphology after LPS treatment detectable in bone marrow-derived macrophages comparing precursor cells isolated from *PARK7*/DJ-1 KO and wildtype mice

**Fig. 5:**
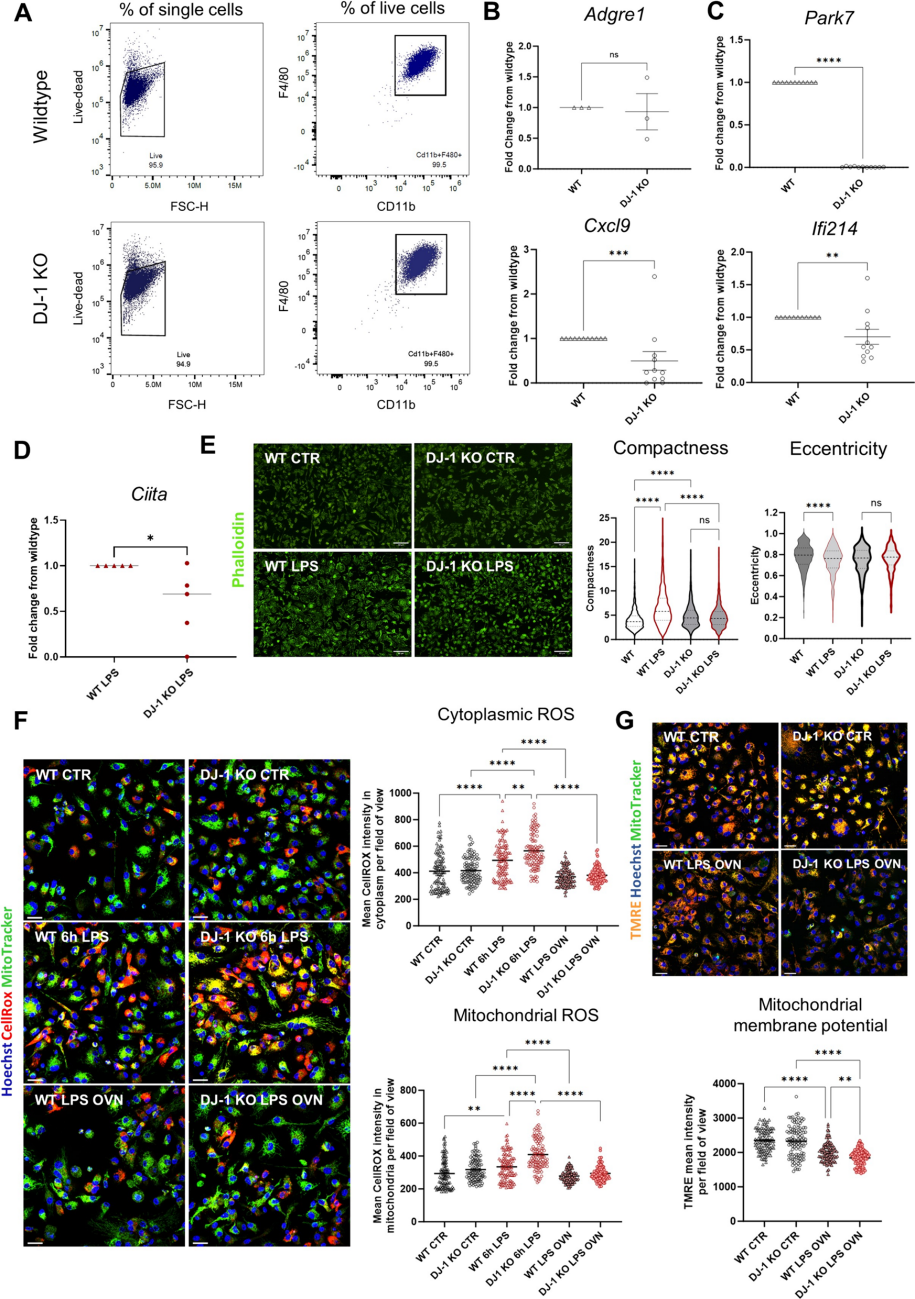
Characterization of *PARK7*/DJ-1-deficient bone marrow-derived macrophages at baseline and after LPS-treatment. **A** Representative gating strategy used to measure percentages of CD11b^+^F4/80^+^ BMDMs among total cells from bone marrow precursors isolated from wildtype (upper panel) or *PARK7*/DJ-1 KO (lower panel) mice (n = 1 mouse per genotype). **B** Gene expression levels of *Adgre1* (F4/80) comparing BMDMs originating from wildtype and *PARK7*/DJ-1 KO mice analyzed by qPCR. Graph shows mean of fold change (wildtype condition set at 1; *Gapdh* as housekeeping gene) ± SEM. Mann–Whitney test, ns: not significant (n = 3 mice per group). **C** Gene expression levels of *Park7, Cxcl9* and *Ifi214* in wildtype and *PARK7*/DJ-1 KO BMDMs analyzed by qPCR. Graphs represent mean of fold change (wildtype condition set at 1; *Gapdh* as housekeeping gene) ± SEM. Mann–Whitney test, ****p < 0.0001, ***p < 0.001, **p < 0.01 (n = 10 mice per group). **D** Gene expression levels of *Ciita* in wildtype and *PARK7*/DJ-1 KO BMDMs under 6 h LPS (100 ng/mL) treatment analyzed by qPCR. Graph represents mean of fold change (wildtype condition set at 1; *Gapdh* as housekeeping gene) ± SEM. Mann–Whitney test, *p < 0.05 (n = 5 mice per group). **E** Representative pictures of wildtype and *PARK7*/DJ-1 KO BMDMs stained with phalloidin showing actin filaments with and without 6 h LPS (100 ng/mL) treatment (left) and corresponding quantification of compactness and eccentricity scores (right). Scale bar: 69 μm. Kruskal–Wallis test for multiple comparisons. ****p < 0.0001, ns: not significant (n = 3 mice per group). **F** Assessment of cytoplasmic and mitochondrial ROS by live imaging. Left: representative images showing labeling of ROS in red and mitochondria in green in BMDMs at baseline, after 6 h LPS and LPS-treatment overnight (OVN). Right: graphs show mean CellROX intensity in cytoplasm and mitochondria per field of view (dots). Kruskal–Wallis test with Dunn’s test for multiple comparisons. ****p < 0.0001, **p < 0.01. **G** Analysis of mitochondrial membrane potential using the specific marker TMRE. Upper panel: representative pictures showing mitochondrial membrane potential (in orange) and mitochondria (in green) in BMDMs at baseline and after overnight treatment with LPS (100 ng/mL). Bottom panel: graph shows mean intensity of TMRE per field of view (dots). Kruskal–Wallis test with Dunn’s test for multiple comparisons. ****p < 0.0001, **p < 0.01

Lastly, to study if the identified murine ex vivo and human in vitro microglial transcriptional profiles under *PARK7*/DJ-1 KO deficiency are specific to brain cells or are also detectable in peripheral myeloid cells, we isolated bone marrow cells from the femurs and tibia of *PARK7*/DJ-1 KO and wildtype mice and differentiated them for 7 days into macrophages [74]. First, we verified that cells lacking *PARK7*/DJ-1 were effectively differentiated into macrophages by analyzing the expression levels of CD11b and F4/80, which indeed did not differ from wildtype cells and represented over 99% of total cells (Fig. 5A). Further, the expression levels of *Adgre1*, coding for F4/80, did not show differences between the two genotypes (Fig. 5B). As detected in microglia at baseline, bone marrow-derived macrophages (BMDMs) from *PARK7*/DJ-1 KO mice showed decreased expression levels of *Cxcl9* and *Ifi214* compared to corresponding cells isolated from wildtype mice (Fig. 5C). Following exposure of BMDMs to LPS (100 ng/mL) for 6 h, cells from *PARK7*/DJ-1 KO mice, as previously demonstrated in microglia, showed decreased expression levels of *Ciita* compared to cells from wildtype mice (Fig. 5D). Notably, in line with the observations we made in mouse brain slices, we detected prominent morphological differences between macrophages originating from *PARK7*/DJ-1 KO and wildtype mice, with KO macrophages showing enhanced compactness at baseline and a less evident amoeboid shape following 6 h of LPS exposure compared to wildtype cells (Fig. 5E). Moreover, we assessed cytoplasmic and mitochondrial ROS levels as well as evaluated the mitochondrial membrane potential with live imaging on BMDMs derived from *PARK7*/DJ-1 KO and wildtype mice. At baseline, there were no difference in both cytoplasmic and mitochondrial ROS levels between the two genotypes (Fig. 5F). However, after 6 h of LPS-treatment (100 ng/mL), ROS levels were significantly higher in macrophages from *PARK7*/DJ-1 KO mice in both compartments. Overnight treatment with LPS led to a decrease in ROS levels both in wildtype and *PARK7*/DJ-1 KO BMDMs compared to the 6-h time point (Fig. 5F). The loss of mitochondrial membrane potential under overnight LPS conditions was more prominent in macrophages from *PARK7*/DJ-1 KO compared to wildtype mice (Fig. 5G), in line with our observations in microglia. These results add on the known scavenging function of DJ-1 during oxidative stress and correspond to previous findings in DJ-1 KO macrophages [1].

Overall, these results are in agreement with the previously characterized microglial transcriptional signatures and morphological adaptations detected in the mouse brain, showing that BMDMs under *PARK7*/DJ-1 deficiency display a reduced IFNrelated signature, both at baseline and after LPS exposure, as well as display similar morphological and ROS level alterations.

## Discussion

At first PD diagnosis, it has been estimated that 30–50% of dopaminergic neurons in the *substantia nigra* have already degenerated [9, 12]. Therefore, for diagnostic and therapeutic perspectives, it is crucial to explore occurrences in prodromal PD phases, the period preceding a PD diagnosis, during which neuroinflammatory processes are already taking place [71, 73]. Thus, in the present study we address this challenge by investigating transcriptional and morphological changes of microglia in a prodromal model of PD, the *PARK7*/DJ-1 KO mouse, at 3–4 months, an age corresponding to adulthood in humans [18], where motor symptoms are not manifested yet [55].

The large majority of studies investigating the functions of DJ-1 in microglia were so far conducted in immortalized microglial cell lines showing that *PARK7*/DJ-1 deficiency sensitizes cells resulting in an exaggerated response to LPS in vitro [42, 75]. In our study, we conducted in vivo and ex vivo analyses, thus achieving a more comprehensive view of the brain, corroborating our findings using translational in vitro models. We previously showed that the downregulation of homeostatic markers and upregulation of classical inflammatory genes represent a typical transcriptional signature of microglia in a LPS model of neuroinflammation [67]. Here, we show that microglia in *PARK7*/DJ-1 KO mice display a distinct response to LPS, specifically characterized by an attenuated inflammatory transcriptional program, which is also recapitulated in human *PARK7*/DJ-1 mutant iPSC-derived microglia (iMG). Among the impaired pathways, we detected a downregulation of genes related to interferon signaling, at baseline and following LPS treatment in both models. Specifically, in microglia isolated from *PARK7*/DJ-1 KO mice, we detected a downregulation of interferon-related genes, both at steady state (e.g. *Cxcl9, Tnfrsf9* and *Ifi214*) and after 6 h (e.g. *Ciita, Ifng, H2-Aa, Oasl2, Gbp8, Ifi213, Ifi27l2a, Tgtp2*) or 24 h (e.g. *Ifitm3, Irf7*) following LPS treatment, aligning with patterns also observed in long-term/chronic stress models [66, 82]. In the DJ-1 mutant iMG, *S100A6, NLRP2* and *ACOD1* were among the most downregulated genes, both at baseline and after LPS treatment. Interestingly, *S100A6* is a gene known to be involved in cellular stress responses and regulation of cytoskeletal functions [81]. *ACOD1* is a key gene involved in immunometabolism coding for an enzyme converting the TCA cycle intermediate cis-aconitate into itaconate, which possesses anti-microbial properties [47]. Of note, *ACOD1* expression is induced by interferon regulating factor 1 (IRF1) [70], thus its decrease can be linked to the impairment of the interferon signaling pathway. Furthermore, *ACOD1* is also an important mediator of mitochondrial ROS generation [29], hence the downregulation of this gene could be a way of adjusting for the loss of DJ-1 in microglia. Previous studies reported excessive inflammatory reaction of DJ-1 deficient astrocytes and microglia to IFN-γ [36] and LPS [41, 46, 79]. Briefly, DJ-1 can bind src-homology 2-domain containing protein phosphatase (SHP-1) and inhibit signal transducer and activator of transcription 1 (STAT-1) signaling in microglia and astrocytes, dampening the inflammatory response initiated via STAT-1 [36]. The downregulation of IFN-related genes at baseline and after LPS-treatment in microglia from *PARK7*/DJ-1 KO adult mice may represent a compensatory mechanism aimed at avoiding an excessive neurotoxic response via the activation of STAT-1 and NFκB. Along these lines, higher levels of glutathione (GSH) have been detected in the medial prefrontal cortex of *PARK7*/DJ-1 KO compared to wildtype mice, which might signify a compensatory mechanism to overcome the loss of the anti-oxidative properties of DJ-1 [11]. Interestingly, changes in GSH metabolism have been observed in various PD patients [3]. This is possibly happening in astrocytes, as those glial cells are known to be important for feeding neurons with precursors for GSH synthesis [54]. In our study, we did not detect upregulation of genes related to the anti-oxidative machinery, neither in murine nor in iMG. However, as we aimed to focus on the effect of the loss of *PARK7*/DJ-1 in microglia, we cannot exclude that compensatory mechanisms took place in surrounding CNS cells. We, therefore, hypothesize that the downregulation of IFN-related genes and upregulation of DNA repair markers, which can be related to tolerogenic mechanisms [25], keep the loss of DJ-1 and oxidative stress in check. However, at a certain stage, representing a “tipping point”, the CNS environment and microglial cells are no longer able to compensate for the loss of *PARK7*/DJ-1 leading to neuroinflammation and neurodegeneration as a consequence of inflammageing and chronic disturbance of the redox balance. This hypothesis is supported by our findings in aged mice where microglia in *PARK7*/DJ-1 KO mice show increased expression levels of pro-inflammatory markers compared to wildtype mice when injected with LPS.

In addition to the attenuated inflammatory response at the transcriptional level, we found discrete morphological adaptations comparing the classical amoeboid shape of microglia after LPS treatment. The understanding of microglial physiology in relation to its morphology is generally limited and the shape of microglia only partly reflect their functions [6]. However, there is consensus on the overarching patterns and correlations between the shape of microglia and certain conditions, such as a less ramified and more compact amoeboid shape is considered an activated microglia reacting to danger or damage signals, whereas a highly ramified structure is considered a feature of surveying homeostatic microglia [78]. Our study found changes in gene ontologies related to cytoskeleton and extracellular matrix reorganization, both in *PARK7*/DJ-1 KO mice and human iPSC-derived DJ-1 mutant microglia. A previous study deeply investigated and described reduced microtubules in DJ-1 deficient neurons [61]. More specifically, *PARK7*/DJ-1 deficient mice exhibited a decreased dendritic complexity with reduced dendritic spine densities in medium spiny neurons [61]. This was found to be a consequence of the downregulation of β-tubulin-III, an important regulator of microtubules, which is regulated by HIF1α. In our study, we found a decreased complexity of microglial processes under steady state conditions in the cortex and *substantia nigra* of *PARK7*/DJ-1 KO mice, which had a more compact shape with thicker and less processes as well as a larger soma. This hyper-ramified morphology was previously observed in microglia in various chronic stress models, where stress was shown to increase microglial internal complexity and degree of branching [32, 65]. Importantly, these observations are in line with the detected impaired interferon-related signatures related to stress conditions at the transcriptional level. As microglial coverage and processes are essential for immunosurveillance, we hypothesize that the reduced complexity of microglia observed in *PARK7*/DJ-1 KO mice at baseline might affect their functions, including altering their communication with other CNS cells and their surveillant features of the brain parenchyma. The pivotal factors influencing process extensions and cell migration involve adhesive interactions with the surrounding extracellular matrix. In this perspective, we found an upregulation of genes related to proteolysis, cell adhesion and extracellular matrix organization both in microglia from *PARK7*/DJ-1 KO mice (e.g. *Adamts13, Elf3, Mefv*) and in human DJ-1 mutant iMG (e.g. *CPA4, ADAM8, CAPN3, HTRA1, CLMP, COL6A3, COL4A1*). These findings suggest that microglia under *PARK7*/DJ-1 deficiency might be more active in the degradation of the extracellular matrix and the secretion of proteins involved in adhering and interacting with the matrix compared to their normal counterparts.

The distinct microglial phenotypic acquisition under *PARK7*/DJ-1 deficiency, both at baseline and following a LPS treatment, might play a role in fostering dopaminergic cell death and amplifying the damage to those vulnerable neurons over time. In fact, a precise balance between an efficient and timely response to infections without compromising the fine-tuned neuronal network is essential for a healthy CNS. Following the impairment of IFN-related pathways that we detected both in the mouse and human models, it would be interesting to further investigate the role of DJ-1 in additional infectious paradigms, such as in viral models. A dampened CNS immune response towards microbial threats under *PARK7*/DJ-1 deficiency could render prodromal PD individuals more susceptible to CNS or systemic infections, thus impacting the brain.

## Conclusions

In summary, our investigations provide a comprehensive transcriptional and morphological analysis of microglia in *PARK7*/DJ-1 KO mice under steady state and inflammatory conditions that we also complement for translational prospects in human *PARK7*/DJ-1 mutant iMG. Our results suggest that microglial cells under *PARK7*/DJ-1 deficiency react differently to an inflammatory stimulus, specifically dampening the interferon-related pathway at the transcriptional level and regulating actin dynamics and cell cytoskeleton affecting their classical amoeboid activated phenotypic state. Furthermore, we detected an impairment of the interferon response at baseline, which is associated with a hyper-ramified morphology representing typical features of microglia under chronic stressful conditions. Taken together, our findings suggest that the underlying chronic oxidative stress associated to the lack of *PARK7*/DJ-1 at baseline affects microglia neuroinflammatory responses, which may play a causative role in PD onset and progression. Further studies are warranted to investigate how the identified molecular and morphological cues underlying *PARK7*/DJ-1 deficiency affect key microglial functions and how they will affect the underlying neuronal network also taking advantage of models of chronic LPS [4] or living microbial infections [33] to assess the triggered neurodegenerative processes.

### Supplementary Information

Supplementary Material 1. Fig. 1. Characterization of wildtype and *PARK7*/DJ-1 KO mouse brains. A) Western blot analysis of DJ-1 and α-actin in whole brain tissue and CD11b^+^ MACS-sorted cells from wildtype and *PARK7*/DJ-1 KO mice. Amount of loaded proteins: wildtype brain tissue: 10 µg; *PARK7*/DJ-1 KO brain tissue: 20 µg; wildtype microglia: 10 µg; *PARK7*/DJ-1 KO microglia: 10 µg. B) Gating strategy used to discriminate lymphocytes (CD45^hi^CD11b^−^ cells) and myeloid cells (CD11b^+^ cells). Among the latter, neutrophils were recognized as Ly6G^+^ cells. From other myeloid cells (Ly6G^−^ cells), we identified border-associated macrophages (BAMs) (CD206^+^Ly6C^−^ cells), monocytes (CD206^−^Ly6C^+^ cells) and microglia (Ly6C^−^CD206^−^ cells). C) Percentages of corresponding cells over CD45^+^ cells extracted from *PARK7*/DJ-1 KO (circles) and wildtype (triangles) mice at baseline and 24 h following LPS treatment. Graphs show mean % of CD45^+^ cells ± SEM. 2-way ANOVA with Tukey’s multiple comparisons, *p < 0.05 (n = 3–5 mice per condition). D) UMAP showing 2931 CD11b^+^CD45^int^ microglial cells from female (pink) and male (green) mice 24 h after LPS treatment (n = 2 mice per group). E) Gene ontology terms corresponding to upregulated genes (adjusted p-value < 0.05, log2FC ≥ 0.5) comparing Cluster 1, 2 or 5 to the other clusters. Dotted line indicates minimum level of significance, i.e. –log10 (adjusted p value = 0.05) = 1.3. F) Analysis of KI67 expression in the cortex of wildtype and *PARK7*/DJ-1 KO mice (positive control in subventricular zone—SVZ). Scale bars: 50–100 μm. G) Gene expression levels of *MKi67* in CD11b^+^CD45^int^ microglial cells analyzed by qPCR (*Gapdh* as housekeeping gene). Bars represent mean ± SEM of analyzed mice (dots). Unpaired t-test, ns: not significant (n ≥ 3 mice).

Supplementary Material 2 Fig. 2. Characterization of CD11b^+^ cells isolated with magnetic beads and validation of identified marker genes discriminating microglia in wildtype and *PARK7*/DJ-1 KO mouse brains. A) Schematic representation of transcriptional analyses of CD11b^+^ microglial cells isolated either from *PARK7*/DJ-1 KO or wildtype mice at baseline and 6 h following LPS treatment. B) Gene expression levels of brain cell markers in CD11b^+^ (blue) and CD11b^−^ (brown) cells analyzed by qPCR. Graphs show relative expression levels of oligodendrocytic (*Mog, Mobp*), astrocytic (*Gfap, Ntsr2*), neuronal (*Tubb3, NeuN*) and microglia (*Gpr34, Olfml3*) markers (*Gapdh* as housekeeping gene). Bars represent mean ± SEM of analyzed male mice (dots). Unpaired t-test, ****p < 0.0001, ***p < 0.001, *p < 0.05 (n ≥ 3 mice). C) Representative picture of CD11b^+^ cells plated and stained with IBA1 antibody (top image) and quantification (n = 3 technical replicates from one mouse) of IBA1^+^ cells (% of total cells based on DAPI staining) (bottom graph). Scale bar: 20 μm. D) Gene expression levels of *Cxcl9*, *Ifi214* and *Mefv* in CD11b^+^ cells isolated from wildtype (n = 7) and *PARK7*/DJ-1 KO (n = 7) mice at baseline analyzed by qPCR (pink dots: females; white dots: males). Graphs represent mean of fold change (wildtype condition set at 1; *Gapdh* as housekeeping gene) ± SEM. Mann–Whitney test, *p < 0.05. E) Gene expression levels of *Cxcl9* and *Ifi214* in primary microglia untreated or treated with IFNγ (50 ng/mL) for 6 h obtained by culturing CD11b^+^ cells isolated from wildtype (n = 4) and *PARK7*/DJ-1 KO (n = 4) mice analyzed by qPCR (pink dots: females; blue dots: males). Graphs represent mean of fold change (wildtype untreated condition set at 1; *Gapdh* as housekeeping gene) ± SEM. Mann–Whitney test, **p < 0.01, *p < 0.05. F) Gene expression levels of *Cxcl9* and *Il6* in CD11b^+^ cells isolated from aged wildtype (n = 5) and *PARK7*/DJ-1 KO (n = 5) mice 6 h following LPS treatment analyzed by qPCR (pink dots: females; blue dots: males). Graphs represent mean of fold change (wildtype LPS condition set at 1; *Gapdh* as housekeeping gene) ± SEM. Mann–Whitney test, **p < 0.01. G) Analysis of mitochondrial membrane potential in microglia at baseline and after LPS treatment (100 ng/mL) overnight using the specific marker TMRE. Graph shows normalized mean intensity of TMRE per field of view (dots) in each group. Kruskal–Wallis test with Dunn’s test for multiple comparisons. ****p < 0.0001 (n = 3 male mice per group). H) Protein expression levels of CD11b in CD11b^+^ cells analyzed by multicolor flow cytometry. Graph shows normalized geometric mean ± SEM of analyzed mice (dots). 1-way ANOVA with Tukey’s multiple comparisons, *p < 0.05 (n = 3 mice).

Supplementary Material 3 Fig. 3. Quality control of human induced pluripotent stem cells, their efficient differentiation into microglia-like cells and differentially expressed genes between *PARK7*/DJ-1 mutant and isogenic control iPSC-derived microglia. A) iPSC colonies stained for nuclei (Hoechst) and specific pluripotent markers, SSEA4, TRA-1–60, OCT4 and SOX2 (upper panel shows isogenic control iPSCs, bottom panel shows *PARK7*/DJ-1 mutant iPSCs). Scale bar: 59 µm. B) *OCT4* and *SOX2* gene expression levels in both isogenic control (iCtrl) and mutant (DJ-1 Mut) iPSC lines analyzed by qPCR. Graphs represent mean of relative expression (iCtrl iPSC set at 1; *Gapdh* as housekeeping gene) (n = 1). C) Gene expression levels of *PARK7* and, specifically, *PARK7* exon 3 in both isogenic control (iCtrl) and mutant (DJ-1 Mut) iPSC lines and iPSC-derived microglia (iMG) by qPCR. Graphs represent mean of relative expression (*GAPDH* as housekeeping gene) ± SEM (n ≥ 1). D) Western blot showing DJ-1 expression, both in isogenic control and mutant lines, in fibroblasts and iPSCs. E) Gene expression levels of microglia homeostatic genes (*GPR34*, *TREM2* and *P2RY12*) in both isogenic control (iCtrl) and mutant (DJ-1 Mut) iPSC lines, in undifferentiated iPSCs and iMG analyzed by qPCR. Graphs represent mean of relative expression (*GAPDH* as housekeeping gene) ± SEM (n ≥ 1). F) Differentially expressed genes (adjusted p-value < 0.01, |log2FC|≥ 1) comparing *PARK7*/DJ-1 mutant and isogenic control iMG under LPS treatment and at baseline (n = 3 replicates of differentiated iMG per condition). Color bar shows log2 Fold Change.

Supplementary Material 4 Fig. 4. Comparison of GO terms associated to genes modulated in DJ-1 deficient microglia under LPS conditions between human and mouse models. A) Venn diagram showing numbers of unique and shared GO terms resulting from gene set enrichment analysis (GSEA) using log fold changes comparing *PARK7*/DJ-1 deficiency with wildtype conditions under LPS conditions from both human and mouse models. B) Dot plot depicting 23 GO terms in common between *PARK7*/DJ-1 mutant iMG and corresponding murine cells under LPS conditions. Color bar shows Normalized Enrichment Score (NES).

Supplementary Material 5.

Supplementary Material 6.

Supplementary Material 7.

Supplementary Material 8.

Supplementary Material 9.

Supplementary Material 10.

Supplementary Material 11.

## Data Availability

The single-cell RNA-sequencing and RNA-sequencing murine data supporting the conclusion of this article have been deposited in the Gene Expression Omnibus (GEO) repository (www.ncbi.nlm.nih.gov/geo/) with accession numbers GSE115571/GSE256105 and GSE254687, respectively. The human RNA-sequencing data has been deposited at the European Genome-phenome Archive (EGA), which is hosted by the EBI and the CRG, under accession number EGAD50000000291. Further information about EGA can be found on https://ega-archive.org "The European Genome-phenome Archive in 2021" [23].

## Abbreviations

BMC: Bone marrow cell
BMDM: Bone marrow-derived macrophage
BP: Biological processes
CNS: Central nervous system
DAPI: 4’,6-Diamidino-2-phenylindole
DAVID: Database for Annotation, Visualization and Integrated Discovery
DEGs: Differentially expressed genes
DJ-1: Protein deglycase-1, Parkinsonism associated deglycase-1 (*PARK7*)
EB: Embryoid bodies
FACS: Fluorescence-activated cell sorting
*Gfap*: *Glial fibrillary acidic protein*
GO: Gene ontology
*Gpr34*: *G-protein coupled receptor 34*
GSH: Glutathione
HBSS: Hanks’ Balanced Salt Solution
IBA1: Ionized calcium-binding adapter molecule 1 (also known as allograft inflammatory factor 1)
IFN-γ: Interferon gamma
iMG: Induced pluripotent stem cell-derived microglia-like cell
iPSC: Induced pluripotent stem cell
IRF1: Interferon regulating factor 1
KO: Knockout
LPS: Lipopolysaccharide
MACS: Magnetic-Activated Cell Sorting
MIC-MAC: Microglia and Immune Cell Morphologies Analyser and Classifier
*Mog*: *Myelin oligodendrocytic glycoprotein*
*Mobp*: *Myelin associated oligodendrocyte basic protein*
*NeuN*: *Neuronal nuclei*
*Ntsr2*: *Neurotensin receptor 2*
OCT4: Octamer-binding transcription factor 4
*Olfml3*: *Olfactomedin like 3*
UMAP: Uniform Manifold Approximation and Projection
*Park7*: *Parkinsonism associated deglycase 7*
PBS: Phosphate buffered saline
PCA: Principal component analysis
PD: Parkinson’s disease
RT-qPCR: Reverse Transcription Quantitative Polymerase Chain Reaction
SEM: Standard error of the mean
SHP-1: Src-homology 2-domain containing protein phosphatase
SOX2: Sex determining region-Y box
SSEA4: Stage-specific embryonic antigen-4
STAT-1: Signal transducer and activator of transcription 1
TGFβ: Tumor growth factor-β
TNF: Tumor necrosis factor
TRA-1-60: Podocalyxin
*Tubb3*: *Tubulin 3*
