# Supplementary figures and images for: PARK7/DJ-1 deficiency impairs microglial activation in response to LPS-induced inflammation
Source: J Neuroinflammation. 2024 Jul 16;21:174. doi: 10.1186/s12974-024-03164-x (PMC11253405; doi:10.1186/s12974-024-03164-x)

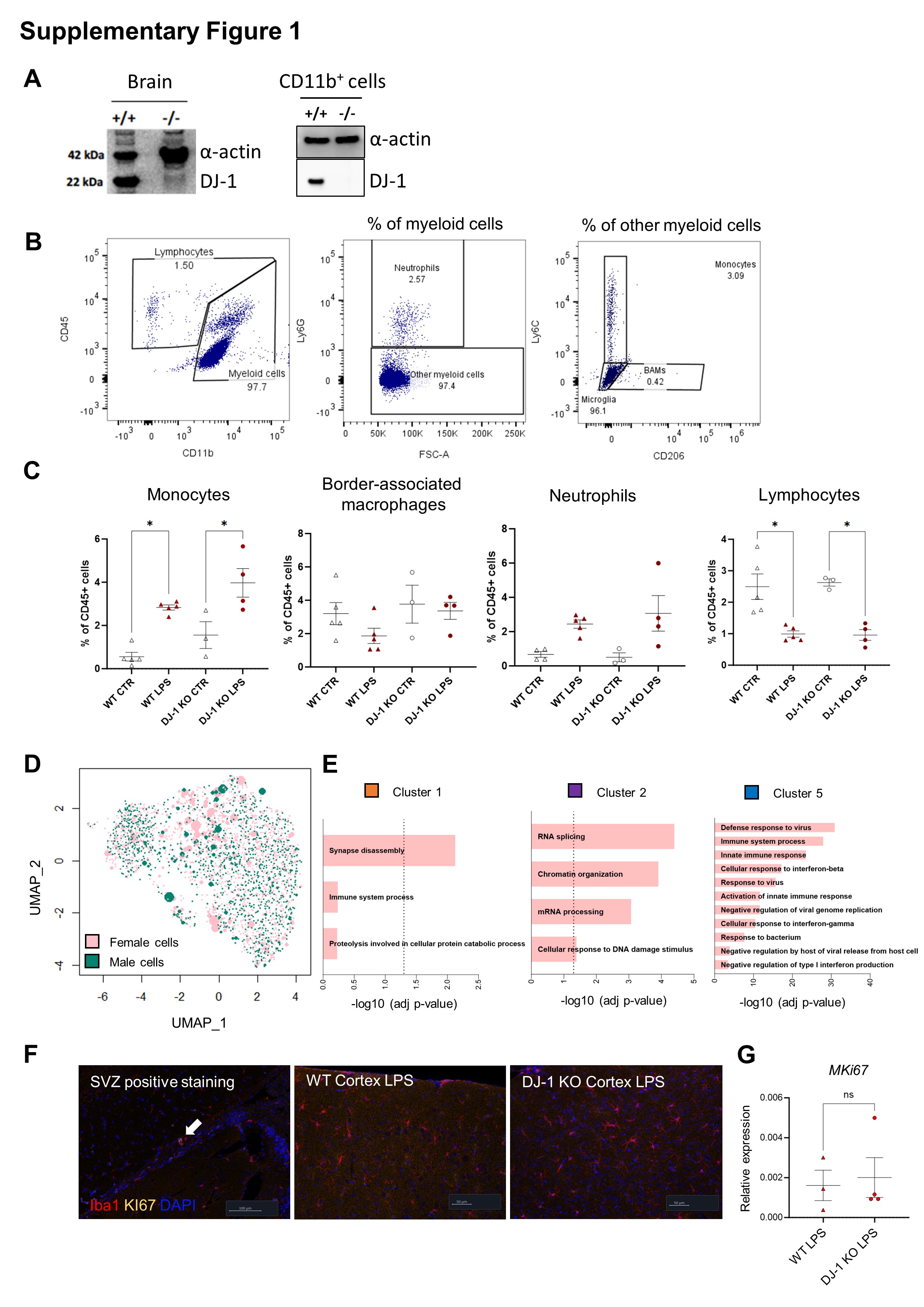

Supplement: Supplementary file 1 — Supplementary Material 1. Fig. 1. Characterization of wildtype and PARK7/DJ-1 KO mouse brains. A) Western blot analysis of DJ-1 and α-actin in whole brain tissue and CD11b+ MACS-sorted cells from wildtype and PARK7/DJ-1 KO mice. Amount of loaded proteins: wildtype brain tissue: 10 µg; PARK7/DJ-1 KO brain tissue: 20 µg; wildtype microglia: 10 µg; PARK7/DJ-1 KO microglia: 10 µg. B) Gating strategy used to discriminate lymphocytes (CD45hiCD11b− cells) and myeloid cells (CD11b+ cells). Among the latter, neutrophils were recognized as Ly6G+ cells. From other myeloid cells (Ly6G− cells), we identified border-associated macrophages (BAMs) (CD206+Ly6C− cells), monocytes (CD206−Ly6C+ cells) and microglia (Ly6C−CD206− cells). C) Percentages of corresponding cells over CD45+ cells extracted from PARK7/DJ-1 KO (circles) and wildtype (triangles) mice at baseline and 24 h following LPS treatment. Graphs show mean % of CD45+ cells ± SEM. 2-way ANOVA with Tukey’s multiple comparisons, *p < 0.05 (n = 3–5 mice per condition). D) UMAP showing 2931 CD11b+CD45int microglial cells from female (pink) and male (green) mice 24 h after LPS treatment (n = 2 mice per group). E) Gene ontology terms corresponding to upregulated genes (adjusted p-value < 0.05, log2FC ≥ 0.5) comparing Cluster 1, 2 or 5 to the other clusters. Dotted line indicates minimum level of significance, i.e. –log10 (adjusted p value = 0.05) = 1.3. F) Analysis of KI67 expression in the cortex of wildtype and PARK7/DJ-1 KO mice (positive control in subventricular zone—SVZ). Scale bars: 50–100 μm. G) Gene expression levels of MKi67 in CD11b+CD45int microglial cells analyzed by qPCR (Gapdh as housekeeping gene). Bars represent mean ± SEM of analyzed mice (dots). Unpaired t-test, ns: not significant (n ≥ 3 mice). [file 12974_2024_3164_MOESM1_ESM.jpg]

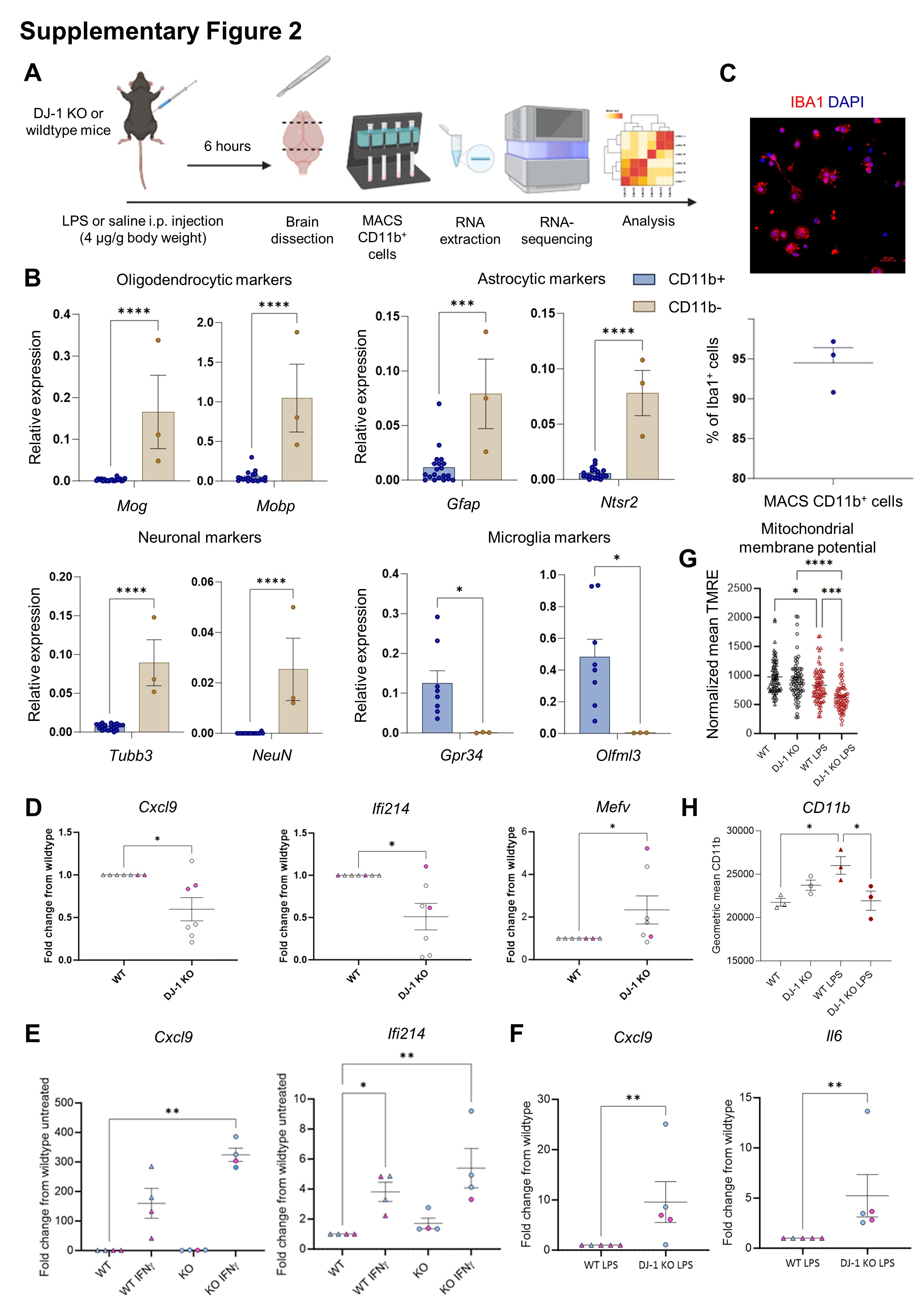

Supplement: Supplementary file 2 — Supplementary Material 2 Fig. 2. Characterization of CD11b+ cells isolated with magnetic beads and validation of identified marker genes discriminating microglia in wildtype and PARK7/DJ-1 KO mouse brains. A) Schematic representation of transcriptional analyses of CD11b+ microglial cells isolated either from PARK7/DJ-1 KO or wildtype mice at baseline and 6 h following LPS treatment. B) Gene expression levels of brain cell markers in CD11b+ (blue) and CD11b− (brown) cells analyzed by qPCR. Graphs show relative expression levels of oligodendrocytic (Mog, Mobp), astrocytic (Gfap, Ntsr2), neuronal (Tubb3, NeuN) and microglia (Gpr34, Olfml3) markers (Gapdh as housekeeping gene). Bars represent mean ± SEM of analyzed male mice (dots). Unpaired t-test, ****p < 0.0001, ***p < 0.001, *p < 0.05 (n ≥ 3 mice). C) Representative picture of CD11b+ cells plated and stained with IBA1 antibody (top image) and quantification (n = 3 technical replicates from one mouse) of IBA1+ cells (% of total cells based on DAPI staining) (bottom graph). Scale bar: 20 μm. D) Gene expression levels of Cxcl9, Ifi214 and Mefv in CD11b+ cells isolated from wildtype (n = 7) and PARK7/DJ-1 KO (n = 7) mice at baseline analyzed by qPCR (pink dots: females; white dots: males). Graphs represent mean of fold change (wildtype condition set at 1; Gapdh as housekeeping gene) ± SEM. Mann–Whitney test, *p < 0.05. E) Gene expression levels of Cxcl9 and Ifi214 in primary microglia untreated or treated with IFNγ (50 ng/mL) for 6 h obtained by culturing CD11b+ cells isolated from wildtype (n = 4) and PARK7/DJ-1 KO (n = 4) mice analyzed by qPCR (pink dots: females; blue dots: males). Graphs represent mean of fold change (wildtype untreated condition set at 1; Gapdh as housekeeping gene) ± SEM. Mann–Whitney test, **p < 0.01, *p < 0.05. F) Gene expression levels of Cxcl9 and Il6 in CD11b+ cells isolated from aged wildtype (n = 5) and PARK7/DJ-1 KO (n = 5) mice 6 h following LPS treatment analyzed by qPCR (pink dots: fema [file 12974_2024_3164_MOESM2_ESM.jpg]

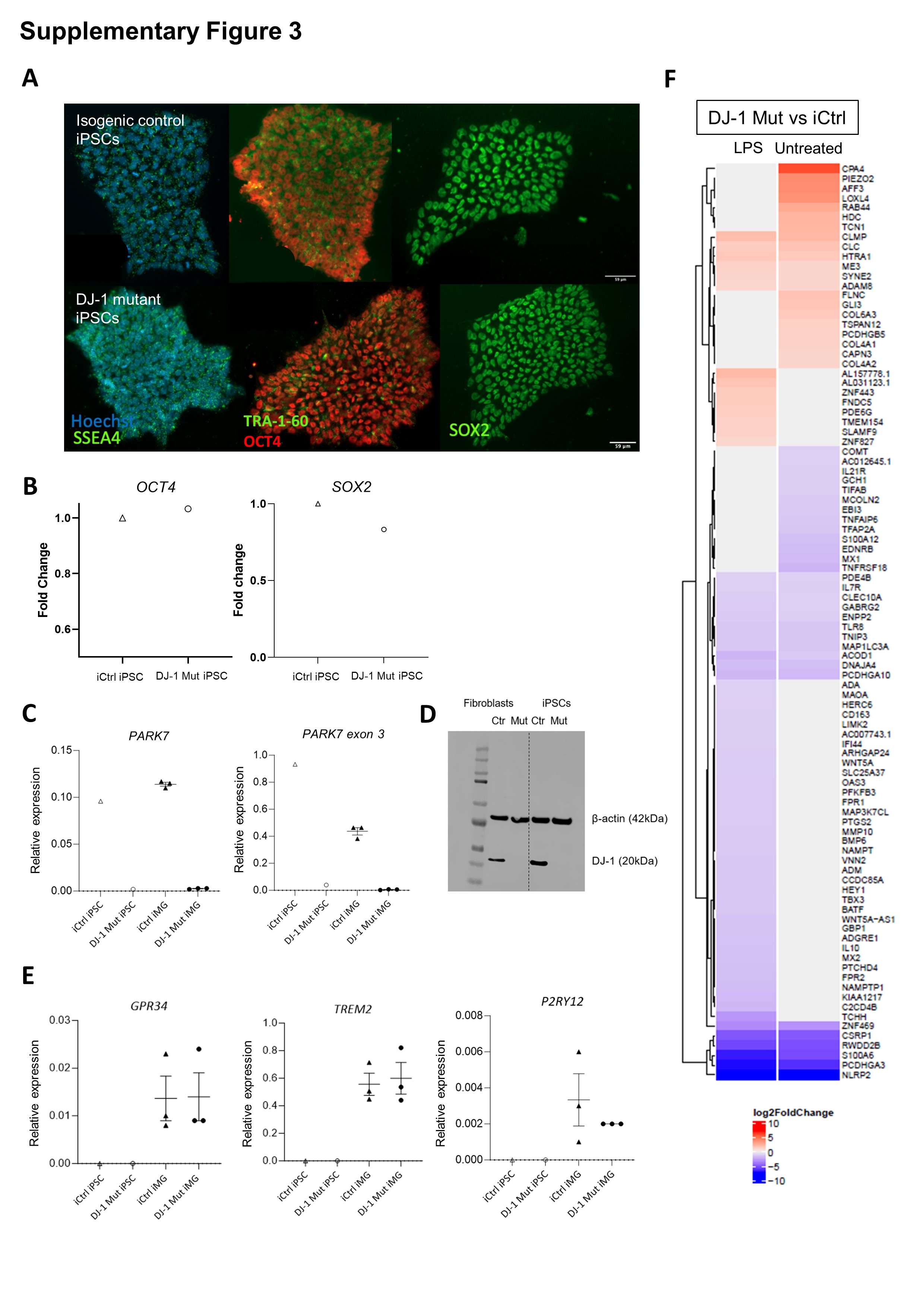

Supplement: Supplementary file 3 — Supplementary Material 3 Fig. 3. Quality control of human induced pluripotent stem cells, their efficient differentiation into microglia-like cells and differentially expressed genes between PARK7/DJ-1 mutant and isogenic control iPSC-derived microglia. A) iPSC colonies stained for nuclei (Hoechst) and specific pluripotent markers, SSEA4, TRA-1–60, OCT4 and SOX2 (upper panel shows isogenic control iPSCs, bottom panel shows PARK7/DJ-1 mutant iPSCs). Scale bar: 59 µm. B) OCT4 and SOX2 gene expression levels in both isogenic control (iCtrl) and mutant (DJ-1 Mut) iPSC lines analyzed by qPCR. Graphs represent mean of relative expression (iCtrl iPSC set at 1; Gapdh as housekeeping gene) (n = 1). C) Gene expression levels of PARK7 and, specifically, PARK7 exon 3 in both isogenic control (iCtrl) and mutant (DJ-1 Mut) iPSC lines and iPSC-derived microglia (iMG) by qPCR. Graphs represent mean of relative expression (GAPDH as housekeeping gene) ± SEM (n ≥ 1). D) Western blot showing DJ-1 expression, both in isogenic control and mutant lines, in fibroblasts and iPSCs. E) Gene expression levels of microglia homeostatic genes (GPR34, TREM2 and P2RY12) in both isogenic control (iCtrl) and mutant (DJ-1 Mut) iPSC lines, in undifferentiated iPSCs and iMG analyzed by qPCR. Graphs represent mean of relative expression (GAPDH as housekeeping gene) ± SEM (n ≥ 1). F) Differentially expressed genes (adjusted p-value < 0.01, |log2FC|≥ 1) comparing PARK7/DJ-1 mutant and isogenic control iMG under LPS treatment and at baseline (n = 3 replicates of differentiated iMG per condition). Color bar shows log2 Fold Change. [file 12974_2024_3164_MOESM3_ESM.jpg]

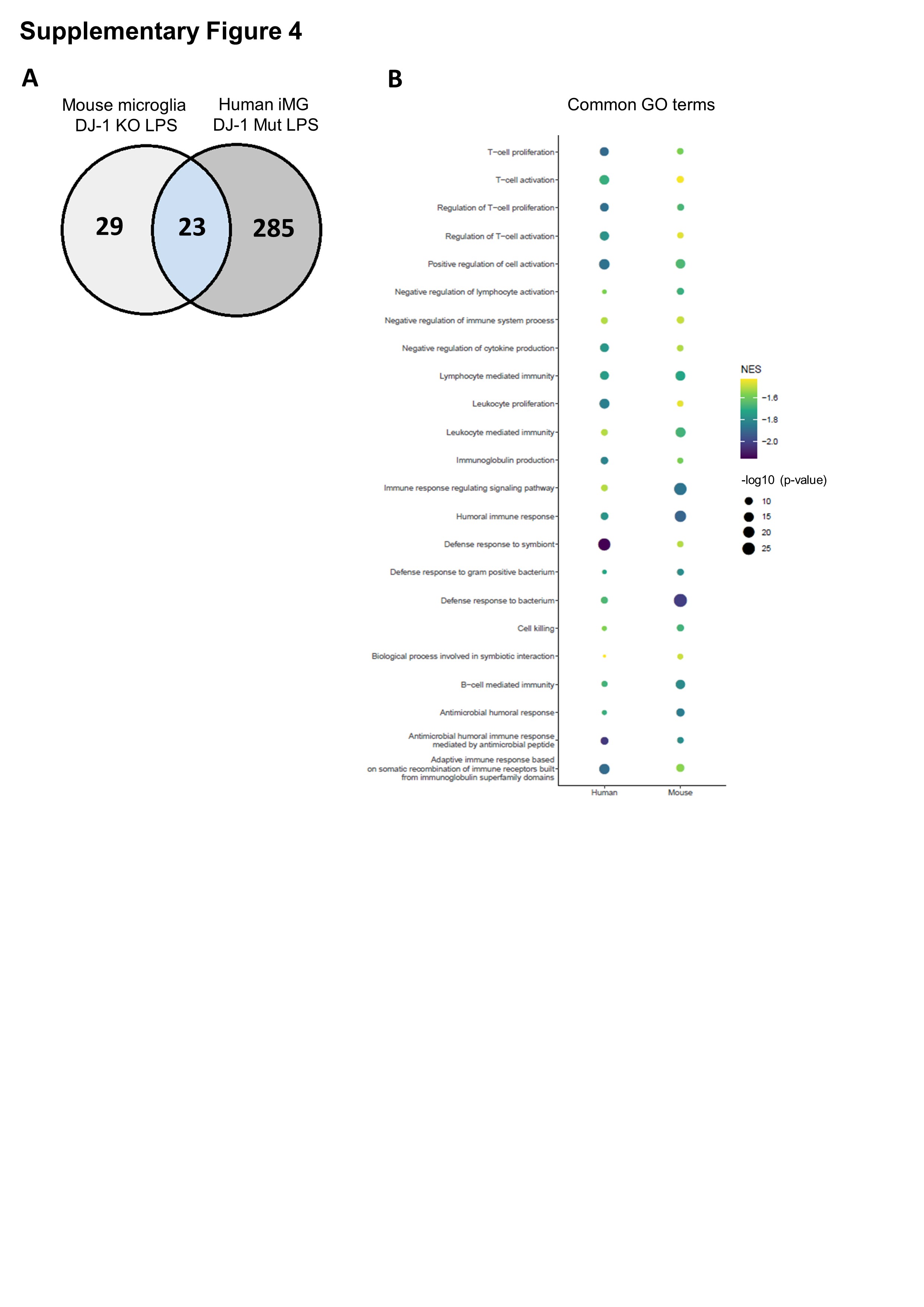

Supplement: Supplementary file 4 — Supplementary Material 4 Fig. 4. Comparison of GO terms associated to genes modulated in DJ-1 deficient microglia under LPS conditions between human and mouse models. A) Venn diagram showing numbers of unique and shared GO terms resulting from gene set enrichment analysis (GSEA) using log fold changes comparing PARK7/DJ-1 deficiency with wildtype conditions under LPS conditions from both human and mouse models. B) Dot plot depicting 23 GO terms in common between PARK7/DJ-1 mutant iMG and corresponding murine cells under LPS conditions. Color bar shows Normalized Enrichment Score (NES). [file 12974_2024_3164_MOESM4_ESM.jpg]
