# Supplementary material for: PARK7/DJ-1 deficiency impairs microglial activation in response to LPS-induced inflammation
Source: J Neuroinflammation. 2024 Jul 16;21:174. doi: 10.1186/s12974-024-03164-x (PMC11253405; doi:10.1186/s12974-024-03164-x)
Supplement: Supplementary file 5 — Supplementary Material 5. [file 12974_2024_3164_MOESM5_ESM.docx]

**Table S1. List of antibodies used for FACS immune-phenotyping, western blotting and immunofluorescence staining.**

| **FACS immune-phenotyping** | | | |
| --- | --- | --- | --- |
| **Antibody** | **Vendor** | **Cat No** | **µl/tube (100 µl)** |
| **CD16/32** | eBioscience | 14016185 | 10 µl |
| **CD11b PerCP_Cy5.5** | eBioscience | 550993 | 5 µl |
| **CD11b PerCP_Cy5.5** | Biolegend | 101227 | 1 µl |
| **F4/80 PE** | Biolegend | 123110 | 5 µl |
| **CD45 FITC** | eBioscience | 11045482 | 1 µl |
| **Ly6G BV785** | Biolegend | 127645 | 2.5 µl |
| **Ly6C PB** | Biolegend | 128014 | 0.5 µl |
| **CD206 APC** | Biolegend | 141708 | 2.5 µl |
| **Goat IgG PE, isotype control** | R&D | IC108P | 5 µl |
| **Western blotting** | | | |
| **Antibody** | **Vendor** | **Cat No** | **Dilution** |
| **Rabbit anti-DJ-1** | Cell Signaling | DP29E25/XP  5933P | 1:1000 |
| **Mouse Anti-Beta-actin (Human)** | Sigma | A1978 | 1:10000 |
| **Mouse Anti-Alpha-actin (Mouse)** | Merck Millipore | MAB1501 | 1:6000 |
| **Anti-Rabbit IgG (H+L) 800** | Cell Signaling | 5151 | 1:5000 |
| **Anti-Mouse IgG HRP** | Merck Millipore | GENA931 | 1:10000 |
| **Anti-Rabbit IgG HRP** | Jackson Laboratories | 111035003 | 1:10000 |
| **Immunofluorescence staining** | | | |
| **Antibody** | **Vendor** | **Cat No** | **Dilution** |
| **Rat Anti-KI67** | Thermo Fisher | 14-5698-82 | 1:1000 |
| **Rabbit Anti-IBA1** | Wako/Fujifilm | 019-19741 | 1:1000 |
| **Chicken Anti-TH** | Abcam | Ab76442 | 1:1000 |
| **Anti-Rat IgG-Alexa Fluor 594** | Thermo Fisher | A-11007 | 1:1000 |
| **Anti-Chicken IgG-Alexa Fluor 488** | Thermo Fisher | A-11039 | 1:1000 |
| **Anti-Rabbit IgG-Alexa Fluor 647** | Thermo Fisher | A-21244 | 1:1000 |
| **Mouse Anti-SSEA4** | Merck Millipore | MAB4304 | 1:1000 |
| **Mouse Anti-TRA-1-60** | Merck Millipore | MAB4360 | 1:1000 |
| **Rabbit Anti-OCT4** | Abcam | Ab19857 | 1:1000 |
| **Mouse Anti-SOX2** | Santa Cruz Biotechnology | Sc-365823 | 1:1000 |
| **Anti-Mouse IgG 488** | Invitrogen | A11001 | 1:1000 |
| **Anti-Rabbit IgG 647** | Invitrogen | A27040 | 1:1000 |
