# Supplementary material for: PARK7/DJ-1 deficiency impairs microglial activation in response to LPS-induced inflammation
Source: J Neuroinflammation. 2024 Jul 16;21:174. doi: 10.1186/s12974-024-03164-x (PMC11253405; doi:10.1186/s12974-024-03164-x)
Supplement: Supplementary file 6 — Supplementary Material 6. [file 12974_2024_3164_MOESM6_ESM.docx]

**Table S2. Growth factor supplements for iPSC-derived microglia-like cells.**

| Differentiation Step | Medium  (Vendor, reference) | Growth factors, cytokines and other supplements |
| --- | --- | --- |
| Fibroblasts | DMEM, Gibco 41965-039 | 10% FBS,  1% Penicillin/Streptomycin |
| iPSCs | mTeSR™ Plus Kit (StemCell technologies, 100-0276) | mTeSR™ 5X Supplement (StemCell Technologies, 100-0276) |
| Embryoid Bodies | mTeSR (StemCell technologies, 100-0276) | mTeSR, 50 ng/ml BMP4 (Invitrogen, PHC9534),  50 ng/ml VEGF (Invitrogen, PHC9394),  20 ng/ml SCF (Miltenyi, 130-096-695) |
| Macrophage Precursor Factory | X-VIVO 15 (Lonza LZBE04-418F) | 100 U/ml Penicillin-Streptomycin (P/S 100x, Gibco 15140-122),  GlutaMax 2 mM (Gibco, 35050-038),  50 µM β-mercaptoethanol (Gibco, 31350-010),  100 ng/ml M-CSF (Invitrogen, PHC9501),  25 ng/ml IL-3 (Invitrogen, PHC0033) |
| Microglia-like Cells | Advanced DMEM (Thermo Fisher, 12634010) | 100 U/ml Penicillin-Streptomycin (P/S 100x, Gibco 15140-122),  GlutaMax 2 mM (Gibco, 35050-038), 1 in 100 N2 (Gibco 17502-001),  50 µM β-mercaptoethanol (Gibco, 31350-010),  10 ng/ml GM-CSF (Peprotech, 300-03-50ug),  100 ng/ml IL-34 (Peprotech, 200-34-100ug) |
