# Supplementary material for: PARK7/DJ-1 deficiency impairs microglial activation in response to LPS-induced inflammation
Source: J Neuroinflammation. 2024 Jul 16;21:174. doi: 10.1186/s12974-024-03164-x (PMC11253405; doi:10.1186/s12974-024-03164-x)
Supplement: Supplementary file 7 — Supplementary Material 7. [file 12974_2024_3164_MOESM7_ESM.docx]

**Table S3. List of mouse and human primers used for qPCR analyses.**

| **Gene** | **PCR primer sequence (5’-3’)** |
| --- | --- |
| ***Gapdh*** | Forward: TGCGACTTCAACAGCAACTC  Reverse: CTTGCTCAGTGTCCTTGCTG |
| ***Adgre1*** | Forward: TCTGGGGAGCTTACGATGGA  Reverse: GAATCCCGCAATGATGGCAC |
| ***Park7*** | - Forward: GCGGCTGCAGTCTTTAAGAAA   Reverse: CCTCCTGGAAGAACCACCAC |
| ***Cxcl9*** | Forward: GCTGTTCTTTTCCTCTTGGGC  Reverse: ATTCCTTATCACTAGGGTTCCTCG |
| ***Ifi214*** | Forward: CTTCCCAACCTATGTCCACCAC  Reverse: GAATGGCTTGGTTCCTTCGGGA |
| ***Ciita*** | Forward: AACTGCGACCAGTTCAGCAA  Reverse: TCTGCTCCAATGTGCTTGAAGA |
| ***Mki67*** | Forward: CCTGCCTGTTTGGAAGGAGTAT  Reverse: TTGGCTTGCTTCCATCCTCA |
| ***Mog*** | Forward: TGCTGACTCTCATCGCACTT  Reverse: CTTCGGTGCAGCCAGTTGTA |
| ***Mobp*** | Forward: CAGACCGGCACGGATGAAAA  Reverse: CCTCCTCAATCTAGTCTTCTGGC |
| ***Gfap*** | Forward: AGAAAGGTTGAATCGCTGGA  Reverse: TCTTGCATGTTACTGGTGGC |
| ***Ntsr2*** | Forward: GGTGAGACACAAGGATGCCA  Reverse: CAGTCCATCCATCATCGGGG |
| ***Tubb3*** | Forward: TGAGGCCTCCTCTCACAAGTA  Reverse: CCGCACGACATCTAGGACTG |
| ***NeuN*** | Forward: ACACACACACTCCATACTGAGG  Reverse: GCTCTGGGCTCTCTGTTTGC |
| ***Gpr34*** | Forward : GGAAAGCTTCAACTCAGTTCCTG  Reverse : TCCATGAGAGGAGCAAAGCC |
| ***Olfml3*** | Forward: TGTTAGACGGCACCCAGAAC  Reverse: CCACTGTTCGGTTTGCCAAG |
| ***Mefv*** | Forward: TCATCTGCTAAACACCCTGGA  Reverse: GGGATCTTAGAGTGGCCCTTC |
| ***Il6*** | Forward: ACCGCTATGAAGTTCCTCTC  Reverse: CTCTGTGAAGTCTCCTCTCC |
| ***GAPDH*** | Forward: TGCACCACCAACTGCTTAGC  Reverse: GGCATGGACTGTGGTCATGA |
| ***OCT4*** | Forward: TCCACTTTGTATAGCCGCTGG  Reverse: TCTCCCCAGCTTGCTTTGAG |
| ***SOX2*** | Forward: TTTGTCGGAGACGGAGAAGC  Reverse: TAACTGTCCATGCGCTGGTT |
| ***PARK7*** | Forward: GCCTGGTGTGGGGCTTGTAA  Reverse: ACCACATCACGGCTACACTG |
| ***PARK7***  ***Exon 3 deletion*** | Forward: ACAGTGTAGCCGTGATGTGG  Reverse: TTCCTGCTCCTTCAGTATCTCC |
| ***GPR34*** | Forward: GCGAACCTGAACTTTAATGCAA  Reverse: GTCGCAACCTTTCACCGTTT |
| ***TREM2*** | Forward: GGGAGTCTGAGAGCTTCGAG  Reverse: TGGGTGGGAAGGGGATTTCT |
| ***P2RY12*** | Forward: TTCAAACCCTCCAGAATCAACAG  Reverse: GTGCACAGACTGGTGTTACC |
